# Yellow Wine Polyphenolic Compounds protect against myocardial ischemia-reperfusion injury in rats by activating Nrf2 nuclear translocation to regulate the balance of mitochondrial fission and fusion

**DOI:** 10.3389/fcvm.2025.1506388

**Published:** 2025-05-23

**Authors:** Lili Xu, Jiedong Zhou, Haifei Lou, Haodi Gu, Haixia Xu, Zuoquan Zhong, Hui Lin, Chengjian Jiang

**Affiliations:** ^1^ Medical Research Center, Shaoxing People’s Hospital, Shaoxing, China; ^2^ School of Medicine, Shaoxing University, Shaoxing, Zhejiang, China; ^3^ Department of Cardiology, Shaoxing People’s Hospital, Shaoxing, Zhejiang, China; ^4^ Department of Pulmonary and Critical Care Medicine, Shaoxing People’s Hospital, Shaoxing, China; ^5^ Department of Cardiovascular, The Affiliated Lihuili Hospital of Ningbo University, Healthy Science Center, Ningbo University, Ningbo, Zhejiang, China

**Keywords:** Yellow Wine Polyphenolic Compounds, ischemia-reperfusion, mitochondrial dynamics, Nrf2, oxidative stress

## Abstract

**Background:**

Myocardial ischemia-reperfusion injury is a pathological phenomenon that occurs after coronary blood flow restoration and poses a threat to patients' lives. Its mechanisms are closely related to oxidative stress and mitochondrial dysfunction. Yellow Wine Polyphenolic Compounds, a dietary polyphenol with significant antioxidant effects, have been shown to offer protection in various cardiovascular diseases. However, their role in MIRI remains under-researched.

**Methods:**

*In vivo* experiments, TTC staining was used to assess myocardial viability, and cardiac ultrasound was employed to measure left ventricular ejection function. Morphological staining and detection of myocardial injury markers were used to evaluate cardiac damage. Transmission electron microscopy was used to observe mitochondrial morphology in myocardial tissue, and ELISA was performed to evaluate the activity of mitochondrial complexes. Adeno-associated virus knockdown was utilized to verify the role of Nrf2. In *in vitro* experiments, confocal microscopy was used to scan mitochondrial morphology in cardiomyocytes and to observe the intracellular localization of the Nrf2 molecule.

**Results:**

TTC staining showed that MIRI significantly increased the infarct size in the left ventricle, whereas pre-treatment with YWPC (Yellow Wine Polyphenol Compound) significantly reduced the infarct area. Cardiac ultrasound demonstrated that YWPC intervention preserved left ventricular ejection fraction. Morphological staining and detection of myocardial injury markers revealed that MIRI caused tissue edema, increased myocardial apoptosis and damage, but YWPC pre-treatment alleviated these injuries. Transmission electron microscopy showed that YWPC reversed the excessive mitochondrial fission caused by MIRI. Immunofluorescence indicated that YWPC significantly promoted Nrf2 nuclear translocation and increased the expression of downstream antioxidant molecules.

**Conclusion:**

YWPC pre-treatment can protect myocardial tissue by reducing the excessive mitochondrial fission induced by MIRI, and Nrf2 mediates these effects.

## Introduction

Myocardial infarction refers to the process of myocardial ischemia and necrosis caused by coronary artery occlusion. Clinically, percutaneous coronary intervention (PCI) with stent implantation or pharmacological thrombolysis is commonly used to achieve myocardial reperfusion therapy. Myocardial ischemia-reperfusion injury (MIRI) refers to the further exacerbation of myocardial damage after the restoration of blood perfusion in ischemic heart disease (1). MIRI is associated with several mechanisms, including a rapid increase in ROS production, calcium overload, inflammatory responses, and mitochondrial dysfunction during vascular reperfusion (2), posing an additional challenge for patients. However, specific pharmacological strategies to prevent and treat MIRI are still lacking. Therefore, it is urgent and meaningful to develop effective drugs targeting oxidative stress and mitochondrial dysfunction to combat MIRI.

Polyphenols are a class of naturally occurring compounds, primarily derived from plants, that possess strong antioxidant properties (3). Numerous studies have demonstrated the protective effects of polyphenols on the human body. For instance, tea polyphenols have been reported to be effective in preventing cancer, diabetes, cardiovascular, and neurological diseases (4). Wine-derived polyphenols are primarily composed of antioxidant compounds such as resveratrol, proanthocyanidins, hydroxycinnamic acids, flavonols, anthocyanins, and hydroxybenzoic acids (5). The protective effect of red wine against coronary heart disease has also been attributed to its polyphenol content (6). Yellow wine polyphenol compounds (YWPC) are dietary polyphenolic constituents extracted from traditional Chinese rice wine, predominantly comprising antioxidant compounds such as gallic acid, epicatechin, quercetin, chlorogenic acid, syringic acid, ferulic acid, and kaempferol (7). We have long focused on studying the cardiovascular effects of YWPC and found that YWPC not only alleviates doxorubicin-induced cardiotoxicity (7, 8), but also has a beneficial effect in slowing down atherosclerosis (9), with its molecular mechanism mainly involving antioxidant activity. However, there is a lack of research on the effects of YWPC in MIRI. Therefore, our goal is to investigate whether YWPC also has a preventive effect on MIRI.

The heart is an energy-intensive organ with a highly active metabolism, where redox reactions are constantly occurring within the mitochondria. There is a mutually reinforcing relationship between mitochondrial dysfunction and oxidative stress (10, 11). Since the occurrence of MIRI is closely associated with oxidative stress and mitochondrial dysfunction, and many of YWPC's effects are based on its antioxidant properties, oxidative stress and mitochondrial function have become the primary focus of our study.

Nfe2l2 (Nrf2), a transcription factor, plays a crucial role in protecting cells against oxidative stress (12). Studies have shown that the absence of Nrf2 exacerbates cardiac injury and dysfunction under exogenous stress conditions (13). Following MIRI, Nrf2 expression is rapidly upregulated, and targeting the promotion of Nrf2 expression can significantly alleviate MIRI. Our previous research has demonstrated that the antioxidant effects of YWPC are mediated through the activation of Nrf2 (8). However, corresponding studies in MIRI are lacking. Therefore, investigating whether the cardioprotective effects of YWPC in MIRI are also mediated by Nrf2 is a research goal worth pursuing.

In this study, we hypothesize that YWPC can effectively improve MIRI by regulating oxidative stress and mitochondrial function. Based on this, we aim to investigate whether these effects require mediation by Nrf2.

## Materials and methods

### Preparation of YWPC

Yellow rice wine was provided by Shaoxing Wine Factory Co., Ltd. The wine is fermented from glutinous rice and wheat koji. The rice-water mixture was combined in a 1:1 ratio, cooked at 125°C for approximately 1 h, and then cooled to room temperature (25°C). After that, Aspergillus was inoculated into the cooked rice to produce saccharifying enzymes, including glucoamylase, glucanase, *α*-amylase, and β-glucosidase. These enzymes break down starch into smaller carbohydrate polymers and sugar monomers, liquefying the rice for more complete fermentation. Fermentation was carried out in sealed red clay containers at 25–33°C. Yellow rice wine was collected when the alcohol content reached 16%–20% (approximately 14–16 days of fermentation).

The extraction of polyphenols was performed by the School of Life Sciences at Shaoxing University. (1). Filter the yellow rice wine through filter paper to remove suspended particles and sediments, obtaining a clear yellow rice wine solution. (2). Solvent mixing: Take a certain volume of yellow rice wine and add 60% ethanol solution in a 1:1 volume ratio. Ethanol can dissolve polyphenols in the yellow rice wine. (3). Extraction: Let the mixture undergo extraction at low temperature for 2 h, stirring occasionally to accelerate the release of polyphenols. (4). Centrifugation: After extraction, place the mixture in a centrifuge and centrifuge at 3,000 rpm for 10 min. Separate the supernatant and retain the polyphenol solution. (5). Rotary evaporation and concentration: Slowly evaporate the ethanol from the polyphenol supernatant using a rotary evaporator, reducing the volume of the solution while keeping the temperature below 40°C to avoid damaging the active polyphenol components. This step yields a crude extract. (6). Drying: Freeze-dry the concentrated crude extract to remove moisture from the solution, obtaining yellow wine polyphenolic compounds. (7). Purification via macroporous resin adsorption: Pass the crude extract through a macroporous resin column to adsorb the polyphenols, followed by ethanol elution. After evaporating the ethanol, purer yellow wine polyphenolic compounds are obtained. (8). Quantitative analysis: Use high-performance liquid chromatography (HPLC) to quantitatively determine the polyphenol content in the extract, verifying the extraction efficiency. The data on compound identification by HPLC has already been reported in our previous research (7).

### Animals and treatment

All experiments were conducted according to the “Guidelines for Animal Care and Use” and approved by the Ethics Committee for Experimental Research at Shaoxing People's Hospital. Healthy male Wistar rats (40–50 days old, 180–210 g) were purchased from Shanghai SLAC Laboratory Animal Co., Ltd. After 1 week of acclimatization, the rats were randomly divided into 8 groups. To evaluate the protective effect of YWPC on MIRI and its dose-response relationship, the following groups were established: Sham group, MIRI group, MIRI + low YWPC group, and MIRI + high YWPC group. For the first 4 weeks, the Sham and MIRI groups were orally gavaged with 2 ml of saline daily. The MIRI + low YWPC group received YWPC solution (3 mg/ml) at a dose of 15 mg/kg/day, while the MIRI + high YWPC group received YWPC solution (6 mg/ml) at a dose of 30 mg/kg/day (8).

On the 29th day, ischemia-reperfusion surgery was performed as follows: Rats were anesthetized with 4% isoflurane, intubated, and mechanically ventilated with 80% oxygen and 20% carbon dioxide. After a left thoracotomy, the heart was exposed in the fourth intercostal space. The left anterior descending (LAD) coronary artery was ligated with a 6–0 suture at 1–2 mm below the left atrial appendage and 0.5 mm adjacent to the pulmonary artery cone. After 30 min, the ligature was released, and reperfusion was maintained for 60 min to establish the ischemia-reperfusion model (14). In the Sham group, the myocardium at the LAD position was only pierced with a surgical suture without ligation. The MIRI, MIRI + low YWPC, and MIRI + high YWPC groups underwent the ischemia-reperfusion surgery.

### Echocardiography

Immediately after reperfusion, cardiac ultrasound examination was performed. Rats were anesthetized with isoflurane, and a VisualSonics Vevo 2100 Imaging System (Toronto, Canada) was used to measure indicators such as left ventricular end-systolic diameter, left ventricular end-diastolic diameter, left ventricular ejection fraction (LVEF), and left ventricular fractional shortening (LVFS).

### Detection of cardiac injury markers

After the ultrasound examination, blood was collected from the tail vein and serum was obtained by centrifugation. ELISA kits (Nanjing Jiancheng, Nanjing, China) were used to measure the concentrations of cardiac injury markers in the serum, including creatine kinase-MB (CK-MB), lactate dehydrogenase (LDH), malondialdehyde (MDA), and cardiac troponin I (cTnI).

### Measurement of infarct size

After completing the ultrasound examination and tail vein blood collection, rats were euthanized with a high dose of isoflurane, and the hearts were harvested. The hearts were sectioned into 5 slices from the mitral valve level to the apex using a surgical knife. The heart slices were placed in 2% 2,3,5-Triphenyltetrazolium chloride (TTC) (Noble Life Technology Co., Ltd., Beijing, China) and incubated in the dark at 37°C for 30 min, with gentle agitation every 5 min to ensure complete staining. After staining, the slices were quickly rinsed in PBS and imaged. The infarct area as a proportion of the left ventricular area was analyzed using Image-ProPlus 6 (Media Cybernetics, Rockville, MD, USA).

### Histopathological examination

The cardiac tissue was fixed in 10% formalin. After undergoing gradient dehydration and embedding in paraffin, 5 µm thick sections were prepared. Hematoxylin and eosin staining was performed using a staining kit (Beyotime, Haimen, China) to assess myocardial morphology. Images of the stained slides were captured using a Leica DM3000 microscope (Leica, Wetzlar, Germany), and image quantification was performed using Image-ProPlus 6 (Media Cybernetics, Rockville, MD, USA).

### TUNEL staining

After deparaffinization with xylene and rehydration through a graded alcohol series, tissue sections were incubated with proteinase K at 37°C for 20 min. They were then incubated with TUNEL working solution (Roche, Basel, Switzerland) at 37°C for 1 h, followed by nuclear staining with DAPI (Beyotime, Haimen, China). Apoptotic positive cells were counted using a Nikon Eclipse Ti-U fluorescence microscope.

### Transmission electron microscopy

The apex of the heart was cut into pieces approximately 1 mm in length and fixed sequentially with 2.5% glutaraldehyde and 1% osmium tetroxide. Following a series of acetone washes and dehydration, the tissue was infiltrated with propylene oxide, embedded in epoxy resin, and sectioned into ultrathin slices. Mitochondrial images were captured using a CS-corrected transmission electron microscope (Titan G2 60–300, FEI, Hillsboro, OR, USA).

### Cell culture and treatment

H9C2 cells were obtained from the Cell Bank of the Chinese Academy of Sciences (Shanghai, China) and cultured at 37°C in a humidified atmosphere with 5% CO2. The growth medium used was Dulbecco's Modified Eagle's Medium (DMEM) containing 10% fetal bovine serum (Gibco, Grand Island, NY). Prior to experimentation, H9C2 cells were cultured until they reached 70%–80% confluence.

The hypoxia-reoxygenation model was established as follows: After removing the complete DMEM medium, cells were washed twice with sterile PBS. The medium was then changed to a serum-free glucose-free medium and incubated in a hypoxic chamber with a low oxygen gas mixture (94% N2, 5% CO2, 1% O2) for 2 h. After 2 h, the medium was switched back to complete medium (10% FBS), and cells were incubated for an additional 4 h under normal conditions.

To further investigate the role of Nrf2 *in vitro*, targeted Nrf2 small interfering RNA (si-Nrf2) (Obio Technology Corp., Ltd, Shanghai, China) was used to interfere with Nrf2 expression, and Western blot analysis confirmed the effective reduction of Nrf2 synthesis by siRNA (Figure 8B).

Five groups were established: Control, H/R, H/R + YWPC, siNC + H/R + YWPC, and siNrf2 + H/R + YWPC. The Control group received no treatment. The H/R group underwent hypoxia-reoxygenation as described above. The H/R + YWPC group was pre-treated with YWPC at a concentration of 50 mg/L for 24 h before undergoing hypoxia-reoxygenation.

For the siNC + H/R + YWPC and siNrf2 + H/R + YWPC groups, 30 nmol of siRNA was transfected into 60% confluent cells using Lipofectamine 3,000 (Invitrogen, USA) in Opti-MEM medium (Gibco, USA). After 48 h, cells were pre-treated with YWPC at the described concentration for 24 h, and then transferred to a hypoxic chamber for subsequent hypoxia-reoxygenation treatment along with the H/R and H/R + YWPC groups.

### Assessment of mitochondrial function

Mitochondria were isolated from fresh rat cardiac tissue or H9C2 cells using the protocol provided with the Mitochondria Isolation Kit (C3606, Beyotime, China). The activities of mitochondrial complexes I, II, III, and IV were determined spectrophotometrically following the manufacturer's instructions with the following specific detection kits: Complex I: Absin Mitochondrial Complex I Activity Assay Kit (abs580238, absin, China), monitored at 340 nm. Complex II: Absin Mitochondrial Complex II Activity Assay Kit (abs580239, absin, China), measured at 605 nm. Complex III: Absin Mitochondrial Complex III Activity Assay Kit (abs580240, absin, China), and Complex IV: Absin Mitochondrial Complex IV Activity Assay Kit (abs580241, absin, China), both quantified at 550 nm.

### Immunofluorescence staining of Nrf2 *in vitro*

H9C2 cells were first fixed with paraformaldehyde and then treated with 0.1% Triton for permeabilization. Following permeabilization, cells were blocked with 10% BSA at 37°C for 30 min. They were subsequently incubated overnight at 4°C with an Nrf2 antibody (#AF0639, Affinity, Jiangsu, China). Afterward, the cells were exposed to Alexa Fluor 594-conjugated goat anti-rabbit secondary antibody (Proteintech, Rosemont, IL, USA) for 1 h. Nuclear staining was performed with DAPI. Confocal images were obtained using a Leica Stellaris microscope (Wetzlar, Germany).

### Detection of cellular ROS and mitochondrial ROS (MitoROS)

Intracellular ROS levels were assessed using a reactive oxygen species assay kit (S0033S, Beyotime, Haimen, China), while mitochondrial ROS (MitoROS) levels were measured with MitoROS staining solution (AAT Bioquest, California, USA), according to the manufacturer's protocols. After removing the cell culture medium, cells were treated with diluted DCFH-DA and MitoROS staining solution. The cells were then incubated at 37°C for 20 min. Following incubation, the cells were washed three times with serum-free cell culture medium to eliminate any residual unincorporated fluorescent dyes. Images were captured using a confocal microscope (Leica Stellaris, Wetzlar, Germany).

### Western blot

Total proteins were extracted from cardiac tissue or H9C2 cells using RIPA lysis buffer (Beyotime, Haimen, China) containing phosphatase inhibitor cocktail II (MedChemExpress, New Jersey, USA). Equal amounts of protein were separated by SDS-PAGE and transferred to a PVDF membrane. The membrane was blocked with 5% non-fat milk for 1 h and then incubated overnight with specific antibodies. Afterward, it was incubated with HRP-conjugated secondary antibodies and developed using enhanced chemiluminescence (Beyotime, Haimen, China). Band quantification was performed using Image-ProPlus 6 (Media Cybernetics, Rockville, MD, USA). The specific antibodies used in this study include: Anti-BAX(ab32503, Abcam, Cambridge, England), anti-BCL2 (ab182858, Abcam, Cambridge, England), anti-cleaved-caspases 3 (ab214430, Abcam, Cambridge, England), anti-phospho-DRP1(ser616) (#AF8470, Affinity, Jiangsu, China), anti-DRP1 (ab184348), anti-MFN1(ab126575, Abcam, Cambridge, England), anti-MFN2(ab124773, Abcam, Cambridge, England), anti-OPA1(ab119685, Abcam, Cambridge, England), Total OXPHOS rodent antibody cocktail (ab110413, Abcam, Cambridge, England), Anti-Bad (ab32445, Abcam, Cambridge, England), Anti-Caspase-3 (ab32351, Abcam, Cambridge, England), Anti-SOD2 (ab68155, Abcam, Cambridge, England), Anti-GPx3 (ab256470, Abcam, Cambridge, England), Anti-Catalase (ab76110, Abcam, Cambridge, England), anti-β-actin (#AF7018, Affinity, Jiangsu, China).

### Assessment of mitochondrial membrane potential

Mitochondrial membrane potential was measured using a JC-1 assay kit (Solarbio, Beijing, China). After the interventions described earlier, 2.5 mmol/L JC-1 dye was added to the H9C2 cell culture plates and incubated in the dark at 37°C for 30 min. Images were captured using a laser scanning confocal microscope (Leica Stellaris, Wetzlar, Germany). Red JC-1 aggregates indicate normal high membrane potential, while green JC-1 monomers signal a loss of mitochondrial membrane potential.

### Mitochondrial morphology imaging

Mitochondria in live H9C2 cells were specifically labeled with 200 nM Mito-Tracker (Beyotime, Haimen, China), while nuclei were labeled with Hoechst. Mitochondrial images were captured using a laser scanning confocal microscope (Leica Stellaris, Wetzlar, Germany).

### Statistical analysis

All data are presented as mean ± SD (standard deviation). Statistical analyses were performed using SPSS 22.0 (SPSS Inc., Chicago, IL), and graphical representations were created with Prism 8 software (GraphPad, San Diego, USA). One-way or two-way ANOVA was used for comparisons among more than two groups, followed by Tukey's *post hoc* analysis. A *p*-value of less than 0.05 was considered statistically significant.

## Result

### Effect of YWPC on cardiac function after MIRI in rats

**Figure 1:**
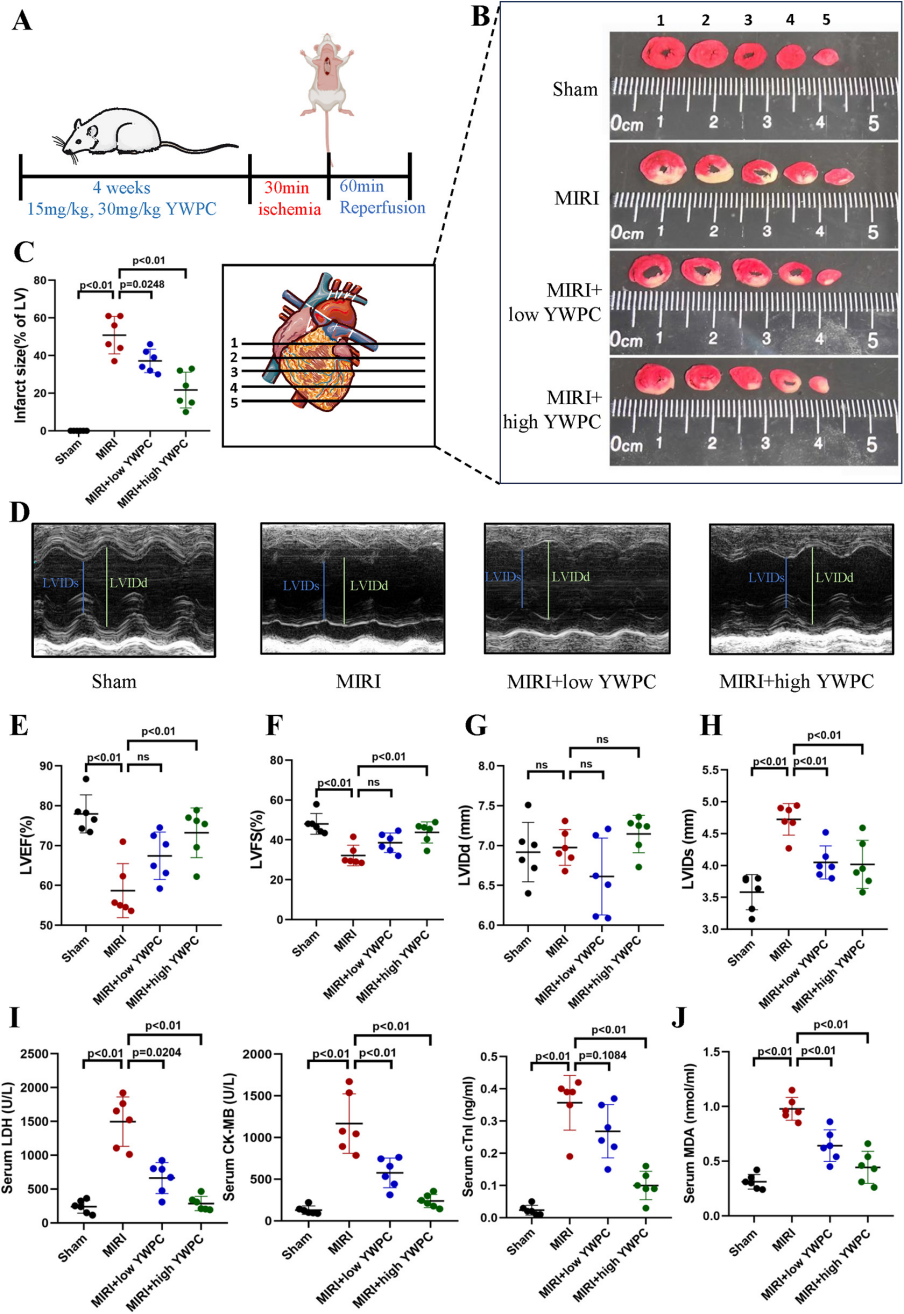
YWPC preconditioning improves cardiac dysfunction induced by ischemia-reperfusion injury. **(A)** Schematic diagram of *in vivo* experiments in this section. **(B,C)** TTC staining shows the infarct size in different groups and the corresponding quantitative data. The images display different transverse sections of the same heart from each group. **(D)** M-mode echocardiography. **(E–H)** M-mode echocardiography measurements of LVEF, LVFS, LVIDd, and LVIDs. **(I)** Serum cardiac injury markers (LDH, CK-MB, and cTnI) measured by ELISA. **(J)** Serum oxidative stress marker (MDA) measured by ELISA. YWPC, Yellow Wine Polyphenolic Compounds; TTC, 2,3,5-Triphenyltetrazolium chloride; LVEF, Left ventricular ejection fraction; LVFS, Left ventricular fractional shortening; LVIDd, Left ventricular internal diameter at end-diastole; LVIDs, left ventricular internal diameter at end-systole; LDH, lactate dehydrogenase; CK-MB, creatine kinase-MB; cTnI, cardiac troponin I; MDA, malondialdehyde. Data presented as mean ± SD. One-way ANOVA was used for comparisons among four groups, followed by Tukey's *post hoc* analysis. ns, Not statistically significant.

To assess the protective effects of YWPC on MIRI and its dose dependency, rats were pretreated with different concentrations of YWPC before establishing the MIRI model (Figure 1A). TTC staining revealed that the heart tissue vitality was intact in the Sham group, with no infarcted areas. In contrast, the MIRI group showed a substantial infarct area in the left ventricle. Preconditioning with a low dose of YWPC (15 mg/kg/d) significantly reduced the left ventricular infarct area compared to the MIRI group. A higher dose of YWPC (30 mg/kg/d) led to an even more pronounced reduction in infarct size compared to the MIRI group (Figures 1B,C). Echocardiography was used to assess cardiac ejection function. As expected, the MIRI group exhibited a significant decrease in left ventricular ejection fraction (LVEF) and left ventricular fractional shortening (LVFS). However, contrary to expectations, the LVEF and LVFS in the low-dose YWPC pretreatment group did not show significant improvement compared to the MIRI group (Figures 1D–F). Surprisingly, the high-dose YWPC pretreatment group showed a significant improvement in both LVEF and LVFS (Figures 1D–F). The left ventricular internal diameter at end-diastole (LVIDd) showed no significant differences among the four groups, indicating that diastolic function was not markedly affected (Figure 1G). Conversely, the left ventricular internal diameter at end-systole (LVIDs) was significantly increased in the MIRI group compared to the Sham group, but it was notably reduced after pretreatment with both low and high doses of YWPC, suggesting that MIRI mainly affects systolic function and YWPC can enhance systolic function (Figure 1H). To further clarify the effects of YWPC on cardiac cell damage, we measured myocardial injury markers LDH, CKMB, and cTnI. We found that all three markers were significantly elevated in the MIRI group. In contrast, pretreatment with either low or high doses of YWPC resulted in a significant reduction in the serum levels of these markers compared to the MIRI group (Figure 1I). Additionally, we evaluated the oxidative stress marker MDA, which was significantly elevated in the MIRI group as expected. However, serum MDA levels were markedly reduced in the YWPC pretreatment groups (Figure 1J).

**Figure 2:**
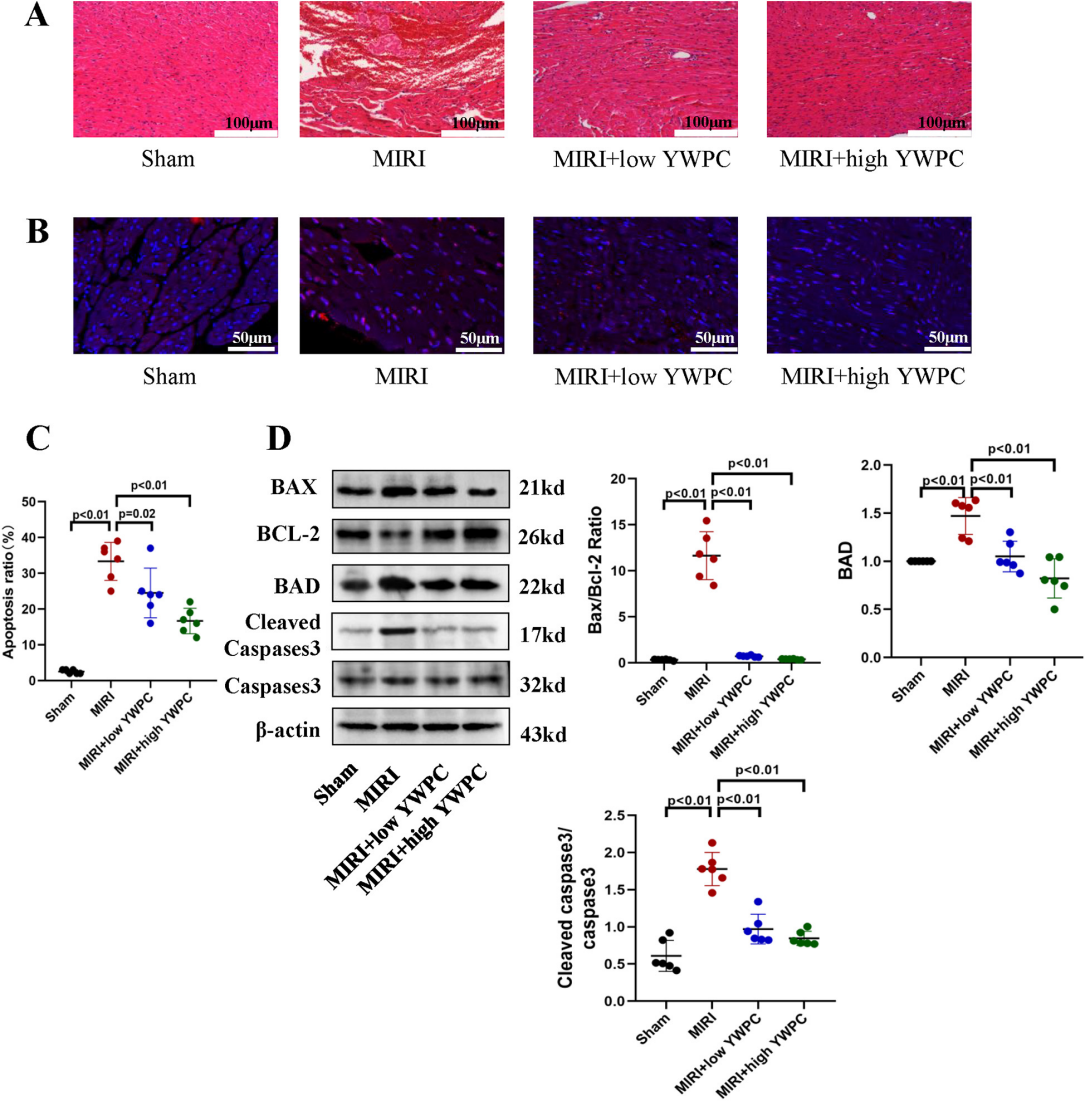
YWPC preconditioning alleviates myocardial edema and apoptosis induced by ischemia-reperfusion injury. **(A)** HE staining used to detect myocardial morphology. **(B,C)** TUNEL assay to assess myocardial apoptosis. **(D)** Western blot analysis of the relative protein expression of BAX, BCL-2, BAD, Cleaved Caspase3 and Caspase3 in myocardial tissue. ns, Not statistically significant. Data presented as mean ± SD. One-way ANOVA was used for comparisons among four groups, followed by Tukey's *post hoc* analysis.

Next, we performed morphological assessments of myocardial tissue. HE staining showed that the MIRI group exhibited myocardial edema and infiltration of inflammatory cells. However, hearts from rats pretreated with both low and high doses of YWPC showed significant improvement in edema (Figure 2A). TUNEL staining revealed extensive apoptosis of myocardial cells following hypoxia-reoxygenation, while YWPC pretreatment significantly reduced the occurrence of apoptosis (Figures 2B,C). Western blot analysis of apoptosis-related proteins BAX, BCL-2, BAD and cleaved caspase-3 showed results consistent with the TUNEL staining. MIRI promoted myocardial cell apoptosis, but YWPC pretreatment inhibited this effect (Figure 2D).

### YWPC regulates myocardial mitochondrial fission-fusion balance and function

**Figure 3:**
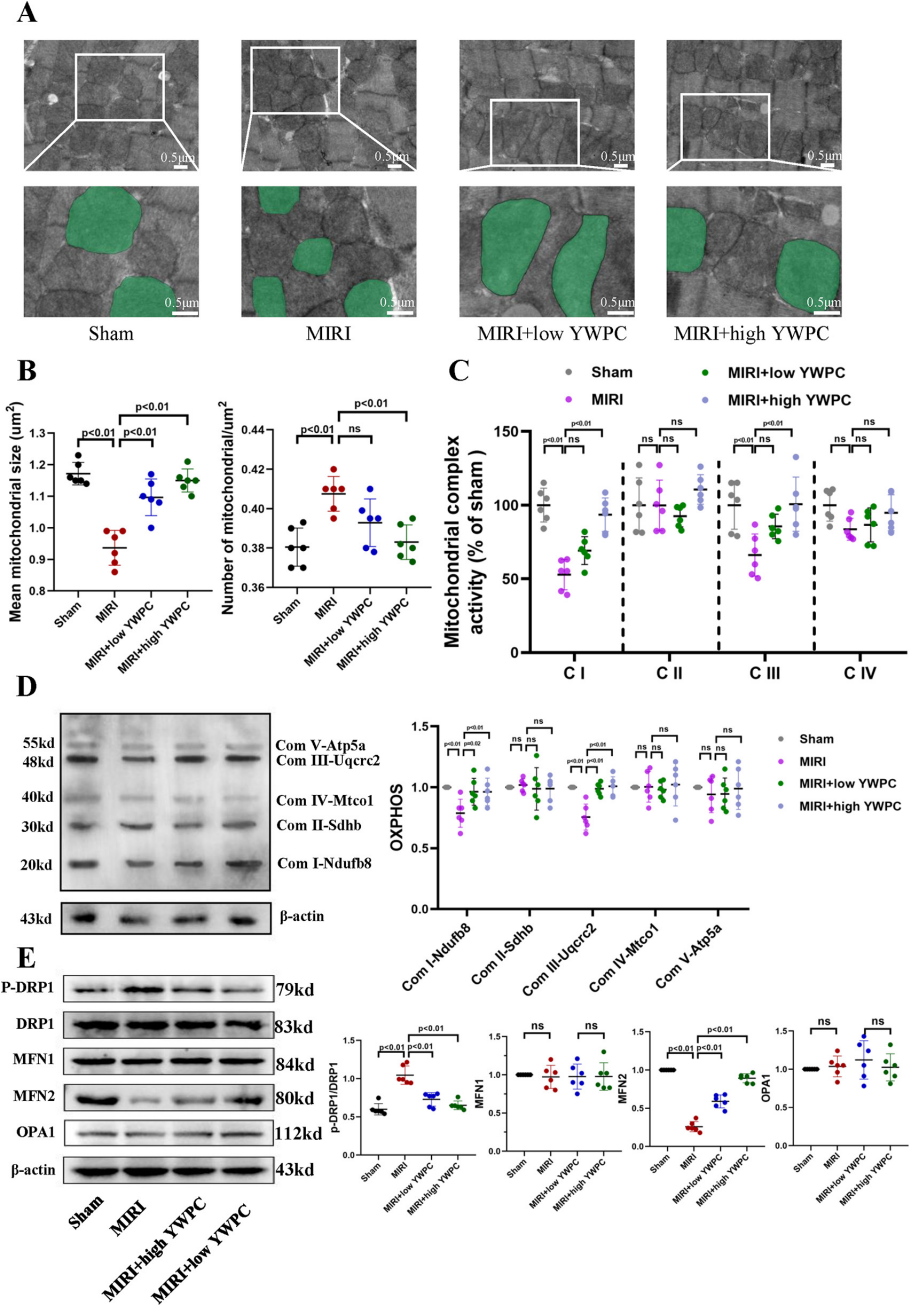
YWPC preconditioning regulates the balance of mitochondrial fission and fusion. **(A)** Representative mitochondrial images obtained by transmission electron microscopy, with representative mitochondria in the bottom images (partial enlargement of the top images) marked with light green pseudocolor to highlight mitochondrial changes. **(B)** Quantitative data of average mitochondrial size and the number of mitochondria per μm^2^. **(C)** Quantitative assessment of mitochondrial respiratory chain complex activity. **(D)** Representative immunoblotting images of mitochondrial OXPHOS proteins in mouse heart lysates (antibodies against Ndufb8, Sdhb, Uqcrc2, Mtco1, and Atp5a1 used as representatives for mitochondrial complex I, II, III, IV, and V). **(E)** Western blot analysis of the relative protein expression of p-DRP1, DRP1, MFN1, MFN2, and OPA1 *in vivo*. OXPHOS, Oxidative phosphorylation; ns, Not statistically significant. Data presented as mean ± SD. One-way ANOVA was used for comparisons among four groups, followed by Tukey's *post hoc* analysis. ns, Not statistically significant.

Given that the heart is an organ with high oxygen consumption and intense metabolism, mitochondrial function is crucial for cardiac health. We used transmission electron microscopy to examine the morphology of mitochondria in myocardial cells and counted the number of mitochondria per unit area. Remarkably, after hypoxia-reoxygenation injury, the average size of mitochondria was significantly reduced, and the number of mitochondria per unit area was notably increased (Figures 3A,B), indicating mitochondrial fission. However, in rats pretreated with both low and high doses of YWPC, the average size of myocardial mitochondria increased significantly compared to the MIRI group, and the number of mitochondria per unit area decreased, suggesting that mitochondrial fission was inhibited. We then assessed the functionality of mitochondrial complexes I, II, III, and IV, finding that MIRI impaired the function of complexes I and III, with no significant effect on complexes II and IV. Both low and high doses of YWPC suppressed the damage to mitochondrial complexes I and III caused by MIRI (Figures 3C,D). Additionally, we used Western blotting to measure mitochondrial fission-fusion dynamics proteins. YWPC was found to reduce the expression of p-DRP and enhance the expression of MFN2, shifting the mitochondrial fission-fusion dynamic balance toward fusion (Figure 3E).

### Nrf2 mediates the protective effects of YWPC on cardiac function

**Figure 4:**
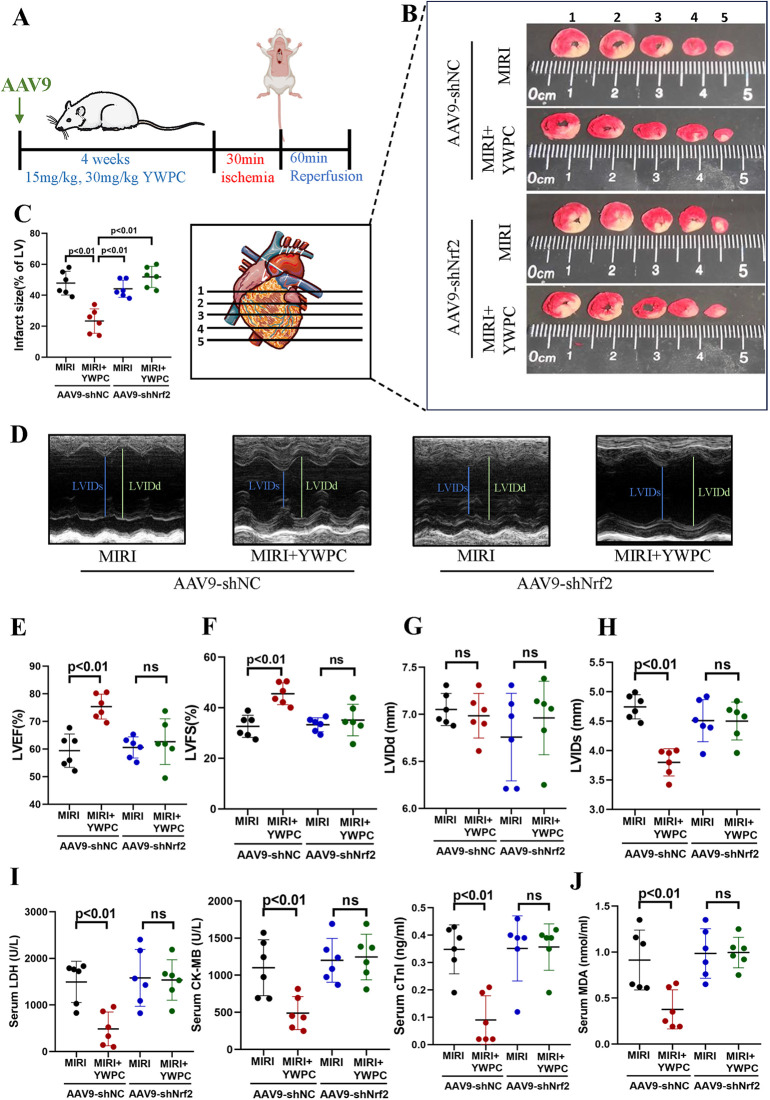
YWPC preconditioning improves ischemia-reperfusion-induced cardiac dysfunction via Nrf2 mediation. **(A)** Schematic diagram of *in vivo* experiments in this section. **(B,C)** TTC staining shows the infarct size in different groups and the corresponding quantitative data. The images display different transverse sections of the same heart from each group. **(D)** M-mode echocardiography. **(E–H)** M-mode echocardiography measurements of LVEF, LVFS, LVIDd, and LVIDs. **(I)** Serum cardiac injury markers (LDH, CK-MB, and cTnI) measured by ELISA. **(J)** Serum oxidative stress marker (MDA) measured by ELISA. YWPC, Yellow Wine Polyphenolic Compounds; TTC, 2,3,5-Triphenyltetrazolium chloride; LVEF, Left ventricular ejection fraction; LVFS, Left ventricular fractional shortening; LVIDd, Left ventricular internal diameter at end-diastole; LVIDs, left ventricular internal diameter at end-systole; LDH, lactate dehydrogenase; CK-MB, creatine kinase-MB; cTnI, cardiac troponin I; MDA, malondialdehyde; ns, Not statistically significant. Data presented as mean ± SD. Two-way ANOVA was used for comparisons among four groups, followed by Tukey's *post hoc* analysis. ns, Not statistically significant.

**Figure 5:**
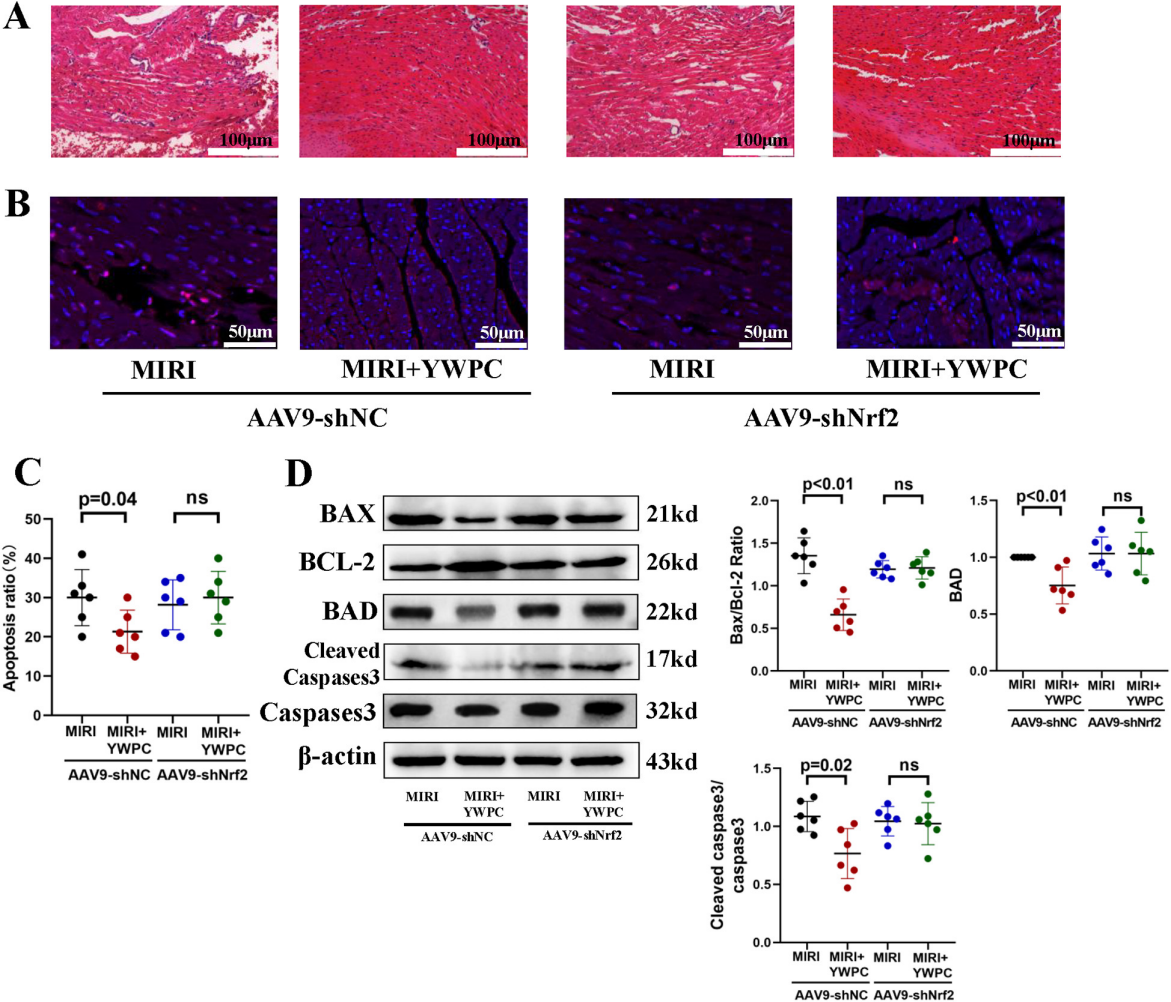
YWPC preconditioning improves ischemia-reperfusion-induced myocardial edema and apoptosis via Nrf2 mediation. **(A)** HE staining used to detect myocardial morphology. **(B,C)** TUNEL assay to assess myocardial apoptosis. **(D)** Western blot analysis of the relative protein expression of BAX, BCL-2, BAD, Cleaved Caspase3 and Caspase3 in myocardial tissue. ns, Not statistically significant. Data presented as mean ± SD. Two-way ANOVA was used for comparisons among four groups, followed by Tukey's *post hoc* analysis. ns, Not statistically significant.

We continued to investigate the target genes through which YWPC exerts its protective effects against hypoxia-reoxygenation injury in myocardial tissue. By injecting AAV9-shNrf2, we achieved knockdown of Nrf2 (Figure 4A), with Western blot confirming a knockdown efficiency of 59.55% (Supplementary Figure S1). TTC staining revealed that in rats injected with AAV9-shNC, YWPC pretreatment still significantly reduced the left ventricular infarct size. However, in rats where Nrf2 was knocked down with AAV9-shNrf2, YWPC pretreatment did not significantly decrease the left ventricular infarct size (Figures 4B,C). Echocardiographic assessment also showed that when Nrf2 was knocked down, YWPC pretreatment did not significantly improve LVEF or LVFS (Figures 4D–F). For the diastolic function indicator LVIDd, there were no significant differences among the four groups (Figure 4G). However, for the systolic function indicator LVIDs, YWPC treatment reduced LVIDs values when Nrf2 was not knocked down, indicating significant improvement in systolic function. This effect was lost when Nrf2 was knocked down (Figure 4H). ELISA tests for myocardial injury markers and oxidative stress indicators similarly showed that YWPC pretreatment could lower serum levels of myocardial injury markers and MDA when Nrf2 was not knocked down, but these protective and antioxidant effects were lost following Nrf2 knockdown (Figures 4I,J). Morphological staining and apoptosis assays also found that YWPC failed to improve myocardial tissue edema and cell apoptosis induced by MIRI when Nrf2 was knocked down (Figures 5A–D).

### Nrf2 mediates the regulation of mitochondria by YWPC

**Figure 6:**
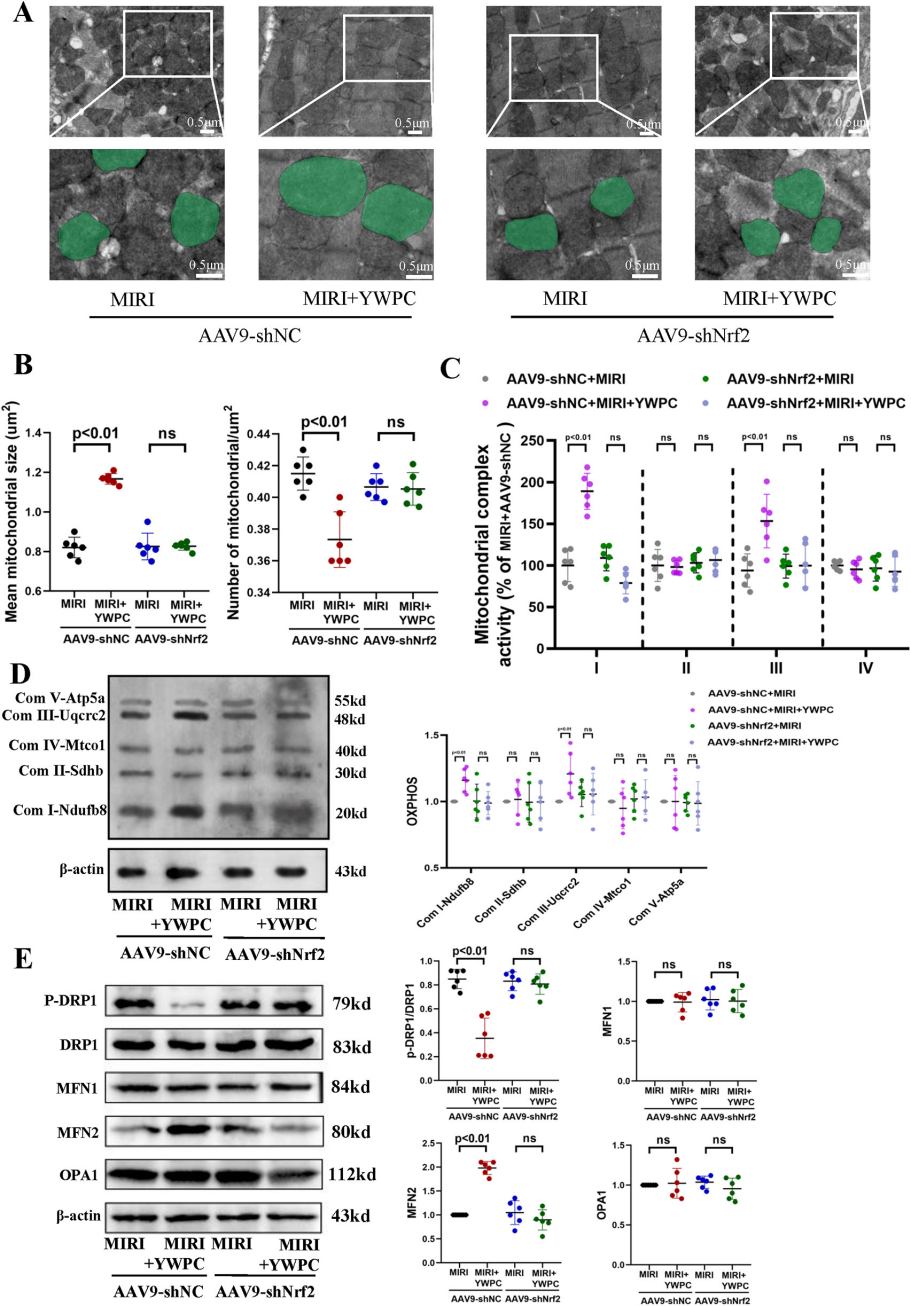
YWPC preconditioning regulates mitochondrial fission-fusion balance via Nrf2 mediation. **(A)** Representative mitochondrial images obtained by transmission electron microscopy, with representative mitochondria in the bottom images (partial enlargement of the top images) marked with light green pseudocolor to highlight mitochondrial changes. **(B)** Quantitative data of average mitochondrial size and the number of mitochondria per μm^2^. **(C)** Quantitative assessment of mitochondrial respiratory chain complex activity. **(D)** Representative immunoblotting images of mitochondrial OXPHOS proteins in mouse heart lysates (antibodies against Ndufb8, Sdhb, Uqcrc2, Mtco1, and Atp5a1 used as representatives for mitochondrial complex I, II, III, IV, and V). **(E)** Western blot analysis of the relative protein expression of p-DRP1, DRP1, MFN1, MFN2, and OPA1 *in vivo*. OXPHOS, Oxidative phosphorylation; ns, Not statistically significant. Data presented as mean ± SD. Two-way ANOVA was used for comparisons among four groups, followed by Tukey's *post hoc* analysis. ns, Not statistically significant.

Based on the findings that YWPC's protective effects require Nrf2 mediation, we further investigated the impact of Nrf2 knockdown on mitochondrial dynamics and function using transmission electron microscopy. We were surprised to find that after Nrf2 knockdown, the regulatory effect of YWPC on mitochondrial fission and fusion became markedly diminished (Figures 6A,B), indicating that Nrf2 is also necessary for YWPC to regulate mitochondrial dynamics in cardiomyocytes. Next, we assessed the function of mitochondrial complexes I, II, III, and IV. As expected, Nrf2 knockdown resulted in a significant reduction in YWPC's protective effects on complexes I and III (Figures 6C,D). Western blot analysis of mitochondrial dynamics-related proteins confirmed these findings, showing that Nrf2 mediates YWPC's effects on mitochondrial fission and fusion (Figure 6E).

### YWPC inhibits H/R-induced apoptosis in H9c2 cells *in vitro*

**Figure 7:**
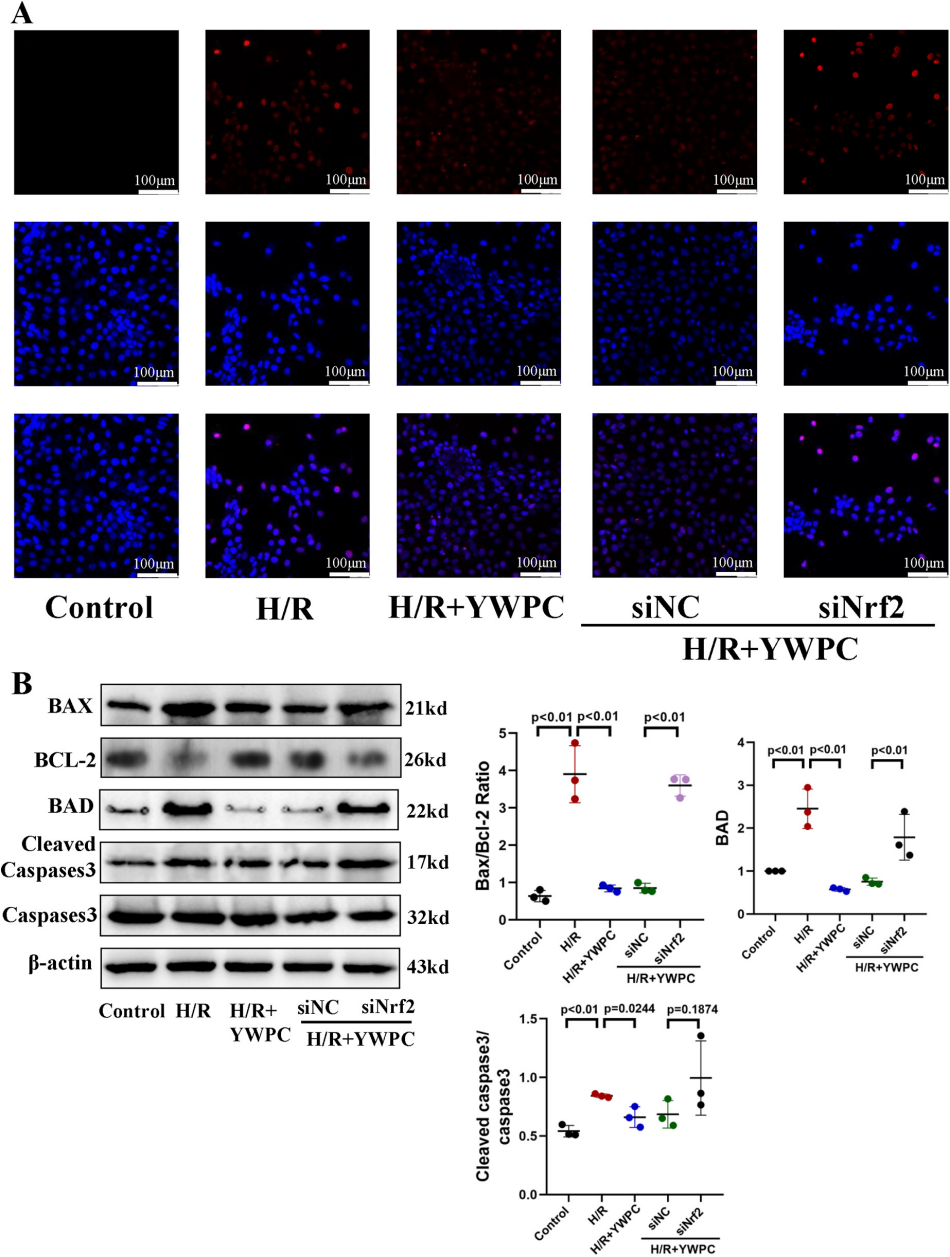
*In vitro*, YWPC reduces apoptosis in cardiomyocytes, mediated by Nrf2. **(A)** TUNEL staining detects cardiomyocyte apoptosis. **(B)** Western blot analysis of the relative protein expression of BAX, BCL-2, BAD, Cleaved Caspase3 and Caspase3 in H9C2 cells. Data presented as mean ± SD. One-way ANOVA was used for comparisons among five groups, followed by Tukey's *post hoc* analysis.

To further confirm the protective effects of YWPC on cardiomyocytes, we cultured H9C2 cells *in vitro* and subjected them to hypoxia/reoxygenation (H/R) treatment. TUNEL staining indicated that H/R significantly increased apoptosis in H9C2 cells, whereas YWPC intervention suppressed apoptosis. To investigate the role of Nrf2 in this process, we used siRNA to interfere with Nrf2 expression. We observed that in the absence of Nrf2, the ability of YWPC to alleviate apoptosis was markedly diminished (Figure 7A). Similarly, Western blot analysis of apoptosis markers showed that YWPC inhibited H/R-induced apoptosis in H9C2 cells *in vitro* and that this effect was mediated by Nrf2 (Figure 7B).

**Figure 8:**
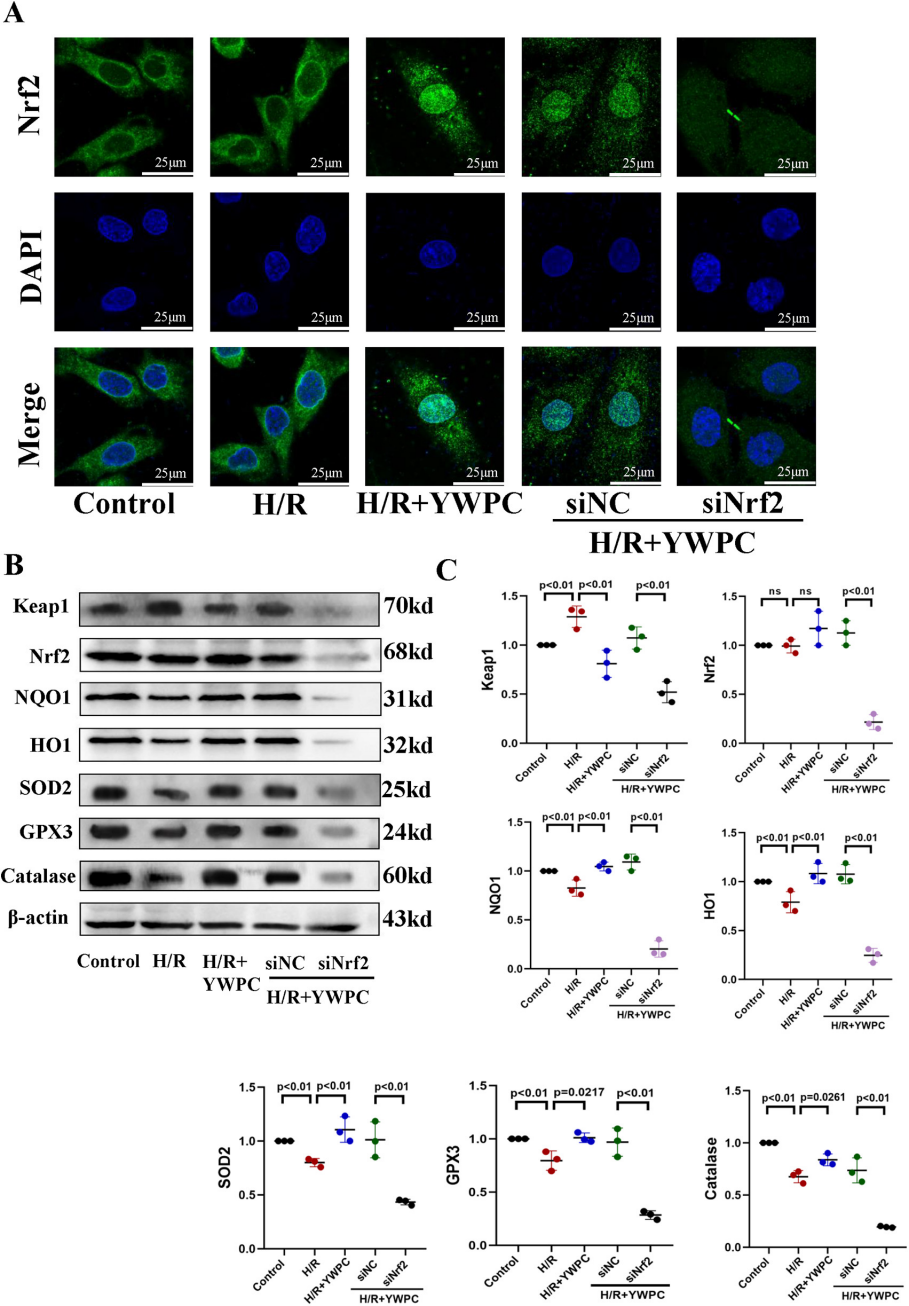
YWPC facilitates Nrf2 nuclear translocation and activation of downstream antioxidant molecule expression. **(A)** Representative images of Nrf2 distribution in H9C2 cells cultured *in vitro*. **(B,C)** Western blot analysis of Keap1, Nrf2, NQO1, HO1, SOD2, GPX3 and Catalase in H9C2 cultured *in vitro*. ns, Not statistically significant. Data presented as mean ± SD. One-way ANOVA was used for comparisons among five groups, followed by Tukey's *post hoc* analysis.

To further explore the specific mechanism through which YWPC activates Nrf2 to exert its antioxidant effects, we performed fluorescence staining for Nrf2. Under physiological conditions, Nrf2 is primarily localized in the cytoplasm. During H/R stress, Nrf2 partially translocates to the nucleus but not sufficiently, whereas YWPC treatment resulted in substantial nuclear translocation of Nrf2 (Figure 8A). Western blot results showed that after YWPC intervention, the expression of downstream antioxidant molecules NQO1, HO1, SOD2, GPX3 and Catalase were significantly increased (Figure 8B). When Nrf2 expression was interfered with, the fluorescence intensity was markedly reduced (Figure 8A), and the expression of downstream antioxidant molecules NQO1, HO1, SOD2, GPX3 and Catalase were also significantly decreased (Figure 8B). To further confirm that YWPC primarily mediates cardioprotection through the Nrf2 pathway and its downstream antioxidant effects, we performed genetic knockdown of HO1, a critical Nrf2-regulated effector. As expected, HO1 knockdown significantly attenuated the anti-apoptotic effects of YWPC in H9C2 cells, reinforcing that Nrf2 and its downstream targets are the primary mediators of YWPC's myocardial protective actions (Supplementary Figure S2A).

### YWPC regulates mitochondrial membrane potential balance and reduces mitochondrial ROS production

**Figure 9:**
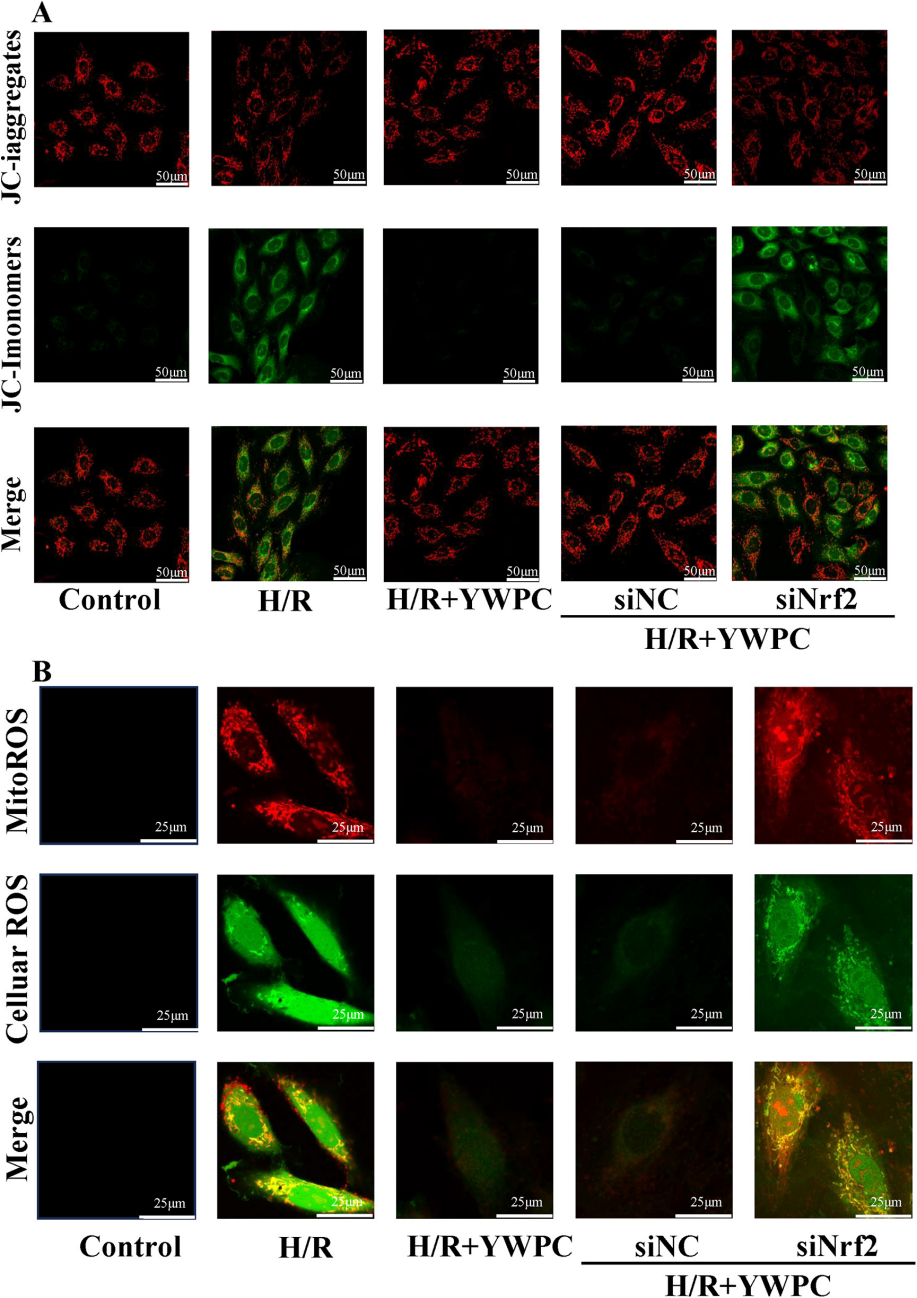
YWPC protects mitochondrial membrane potential and reduces mitochondrial-derived ROS production. **(A)** JC-1 assay detects mitochondrial membrane potential in H9C2 cells. **(B)** Representative images of MitoROS-stained mitochondrial ROS (red fluorescence) and DCFH-DA-stained cellular ROS production (green fluorescence), as well as co-localization (yellow fluorescence).

Based on the importance of mitochondrial function for cardiomyocytes, we evaluated the changes in mitochondrial membrane potential using the JC-1 reagent kit *in vitro*. Normal H9C2 cells exhibit red fluorescence in their mitochondria, indicating a normal mitochondrial membrane potential. However, after H/R treatment, the fluorescence shifts to green, indicating a decrease in mitochondrial membrane potential. After intervention with YWPC, the membrane potential was observed to return to nearly normal levels. However, when Nrf2 expression was disrupted, the protective effect of YWPC on mitochondrial membrane potential was lost (Figure 9A).

Since mitochondria are one of the organelles that produce reactive oxygen species (ROS), we also labeled mitochondrial-derived ROS (mitoROS) (red fluorescence) and cellular ROS (green fluorescence). Surprisingly, we found a strong co-localization of mitoROS and cellular ROS, indicating that under H/R stress, ROS in the cell mainly originates from mitochondria. YWPC intervention significantly reduced mitoROS. However, when Nrf2 expression was disrupted, the antioxidant effect of YWPC was significantly diminished (Figure 9B).

### YWPC regulates mitochondrial fission-fusion dynamics in H9c2 cells

**Figure 10:**
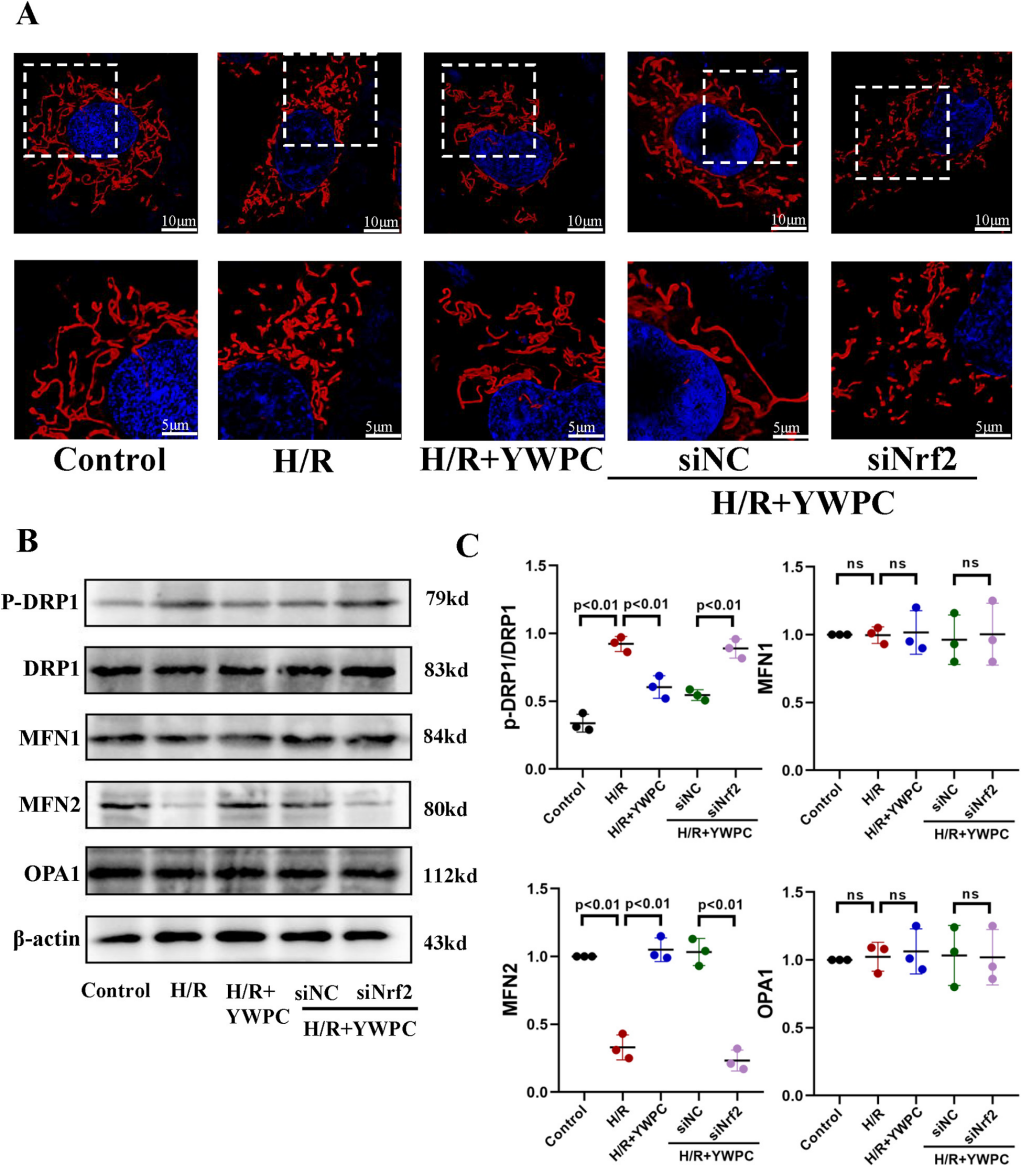
*In vitro*, YWPC regulates mitochondrial fission-fusion balance in H9C2 cells, mediated by Nrf2. **(A)** Morphological images of mitochondria in H9C2 cells under different intervention conditions. **(B,C)** Western blot analysis of the relative protein expression of p-DRP1-S616, DRP1, MFN1, MFN2, and OPA1 in H9C2 cells cultured *in vitro*. ns, not significant. Data presented as mean ± SD. One-way ANOVA was used for comparisons among five groups, followed by Tukey's *post hoc* analysis. ns, Not statistically significant.

Mitochondria were labeled using mitochondrial probes and imaged under confocal microscopy. In cultured H9C2 cells, mitochondria in normal cardiomyocytes appear elongated, while H/R treatment significantly promotes mitochondrial fission, resulting in short, rod-like structures and an increased number of mitochondria. However, YWPC intervention restores the mitochondria to an elongated form (Figure 10A). Analysis of mitochondrial fission-fusion dynamics-related proteins showed that YWPC decreased the expression of p-DRP and promoted the expression of MFN2, shifting the balance of mitochondrial fission-fusion dynamics towards fusion (Figures 10B,C). However, in the absence of Nrf2 expression, the effect of YWPC in inhibiting mitochondrial fission in H9C2 cells was significantly weakened (Figure 10A), and there were no significant differences in the expression of p-DRP1 and MFN2 (Figures 10B,C).

## Discussion

Increasing research indicates that antioxidants play a crucial protective role in cardiac function (15). In this study, we demonstrated that YWPC, a potent antioxidant, significantly promotes Nrf2 nuclear translocation, thereby activating the expression of downstream antioxidant enzymes including NQO1, HO1 and SOD to restore redox homeostasis. Given the reciprocal interplay between oxidative stress and mitochondrial dysfunction, YWPC treatment normalized impaired mitochondrial dynamics (fusion/fission), enhanced the activity of respiratory chain complexes, and markedly attenuated mitoROS production. This reduction in mitoROS consequently diminished total intracellular ROS levels, ultimately alleviating myocardial cell apoptosis induced by MIRI.

Clinically, the treatment of myocardial infarction primarily involves coronary stent implantation or thrombolysis to restore blood supply to the ischemic area, which inevitably leads to ischemia-reperfusion injury. The mechanisms of ischemia-reperfusion injury include the generation of oxygen free radicals, calcium overload, inflammatory responses, and mitochondrial energy metabolism disorders (2). Various pretreatment strategies can mitigate the damage caused by ischemia-reperfusion. Literature reports that pretreatment with the NLRP3 inhibitor INF195 can significantly inhibit IL-1β production, thereby reducing inflammation and significantly decreasing the myocardial infarction area (16). Clinical studies have reported that allopurinol pretreatment inhibits the activation of the xanthine oxidase system and the production of reactive oxygen species, improving blood flow restoration in PCI patients (17). Not only for the heart, but mild hypothermia has also been reported to effectively reduce oxidative stress and inflammation, alleviating liver ischemia-reperfusion injury (18). Therefore, adequate pretreatment, such as antioxidant therapy, before reperfusion is crucial. YWPC, a dietary polyphenol with strong antioxidant properties, has been extensively studied by our team. Our current research shows that pretreatment with YWPC significantly reduces the infarct area ratio caused by MIRI, decreases cardiomyocyte apoptosis and damage. Additionally, MDA levels, an oxidative stress marker, were significantly lower in the YWPC pretreatment group, indicating that the cardioprotective effects of YWPC are closely related to its antioxidant properties.

As one of the organs with extremely high oxygen consumption, mitochondrial homeostasis plays a crucial role in cardiac function. Mitochondrial fission and fusion are key mechanisms for maintaining mitochondrial homeostasis and are vital for proper mitochondrial function. Mitochondrial fusion ensures optimal mitochondrial activity by allowing the exchange of contents between mitochondria and forming a network that aids in energy transfer to the cell core (19). The fusion process is mainly mediated by molecules such as OPA1, MFN1, and MFN2. Mitochondrial fission is important for maintaining mitochondrial number and proper distribution within cells, and while mitochondrial fission is generally beneficial, particularly in cells requiring high energy (20), excessive fission is often observed under stress conditions (21). Numerous studies have reported that excessive mitochondrial fission is associated with diabetic cardiomyopathy and cardiac ischemia-reperfusion injury (22, 23). Excessive fission leads to changes in mitochondrial morphology and architecture, affecting mitochondrial function and stability, triggering mitochondrial-mediated apoptotic pathways, and ultimately leading to cell death (24). Moreover, in pathological conditions, excessive fission is an upstream event for ROS production (25). In our study, we found that MIRI leads to excessive mitochondrial fission in cardiomyocytes. By examining mitochondrial respiratory chain complexes, we observed impaired respiratory chain function, accompanied by a significant increase in intracellular ROS. Fluorescent co-localization studies revealed that intracellular ROS mainly originate from mitochondria. Intervention with YWPC reversed excessive mitochondrial fission and repaired the function of mitochondrial respiratory chain complexes. Additionally, we observed that YWPC provided significant protection for mitochondrial membrane potential and reduced intracellular ROS. We also examined mitochondrial dynamics-related proteins and found that, as we hypothesized, when H/R stress disrupted the balance of mitochondrial fission and fusion, YWPC appropriately reversed the increase in p-DRP1 and upregulated MFN2 expression, maintaining mitochondrial homeostasis, which is crucial for maintaining cellular redox balance.

Nuclear factor erythroid 2-related factor 2 (Nfe2l2, Nrf2) is a crucial transcription factor that regulates cellular redox balance. Nrf2 protects cells from oxidative damage by binding to antioxidant response elements (ARE) and regulating the expression of a range of antioxidant proteins and detoxifying enzymes (26). Under normal conditions, Nrf2 is bound to Keap1 protein in the cytoplasm and is ubiquitinated and degraded. When cells are exposed to oxidative stress, Nrf2 dissociates from Keap1, translocates to the nucleus, and activates the expression of antioxidant genes such as NQO1 and HO1 (26). The relationship between Nrf2 and the heart is extremely close, with reports indicating its protective role in disease models such as doxorubicin-induced cardiomyopathy (8, 27) and diabetic cardiomyopathy (28). For MIRI, numerous studies have shown that activation of Nrf2 can significantly reduce oxidative stress and protect cardiomyocytes (29–31). In our study, we found that YWPC promotes the translocation of Nrf2 from the cytoplasm to the nucleus and enhances the expression of downstream molecules NQO1, HO1, SOD, GPX3 and Catalase, which is crucial for resisting oxidative stress. When we knocked down or interfered with Nrf2 expression, we observed that the cardioprotective effect of YWPC was greatly diminished, further validating our hypothesis.

The limitations and advantages of this study are also worth discussing. Firstly, Our experimental findings were exclusively derived from male rats, and their applicability to female rodents remains to be validated. More critically, given the physiological differences between rodent and human cardiovascular systems, our conclusions must be further validated through rigorously designed clinical trials. However, using rats as study subjects allows for extremely strict control over variables such as sex, age, genetic background, diet, and daily exercise intensity, which is crucial for studying mitochondrial dynamics and cardiac function. This ensures the stability and reproducibility of the research findings. Secondly, in addition to oxidative stress, the pathogenesis of MIRI is also related to factors such as calcium overload and activation of inflammatory pathways, which interact with each other. This project currently only investigates the effects of YWPC on oxidative stress and mitochondrial fission-fusion dynamics. Thus, further research on the regulatory effects of YWPC on calcium overload and inflammatory pathways is also necessary, and this has become one of our next research goals.

## Data Availability

The original contributions presented in the study are included in the article/Supplementary Material, further inquiries can be directed to the corresponding authors.
