# Supplementary material for: Yellow Wine Polyphenolic Compounds protect against myocardial ischemia-reperfusion injury in rats by activating Nrf2 nuclear translocation to regulate the balance of mitochondrial fission and fusion
Source: Front Cardiovasc Med. 2025 May 23;12:1506388. doi: 10.3389/fcvm.2025.1506388 (PMC12141274; doi:10.3389/fcvm.2025.1506388)
Supplement: Supplementary file 1 [file Datasheet1.pdf]

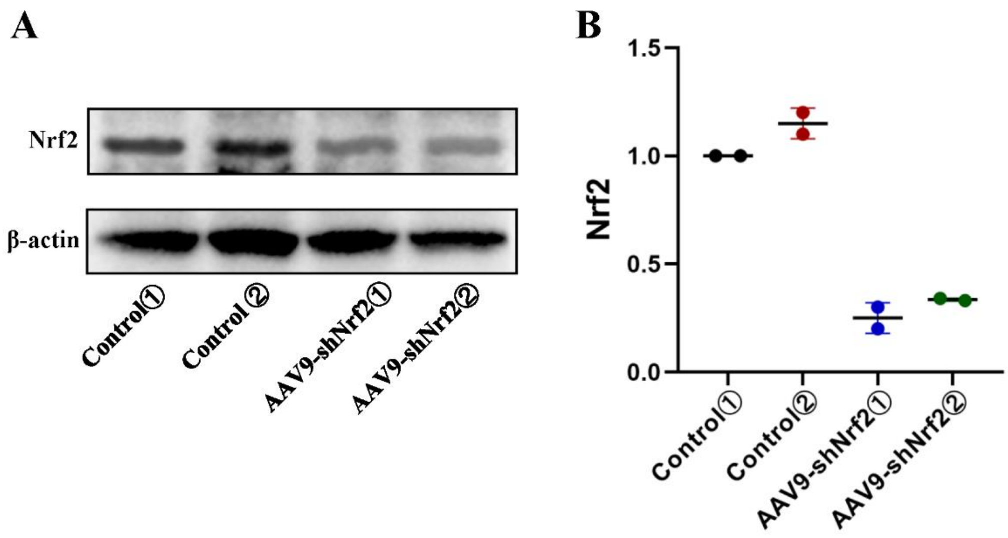

Supplementary Figure 1: Verification of Nrf2 knockdown efficiency using adeno-associated virus. (A-B) Western blot analysis of Nrf2 expression and quantification.

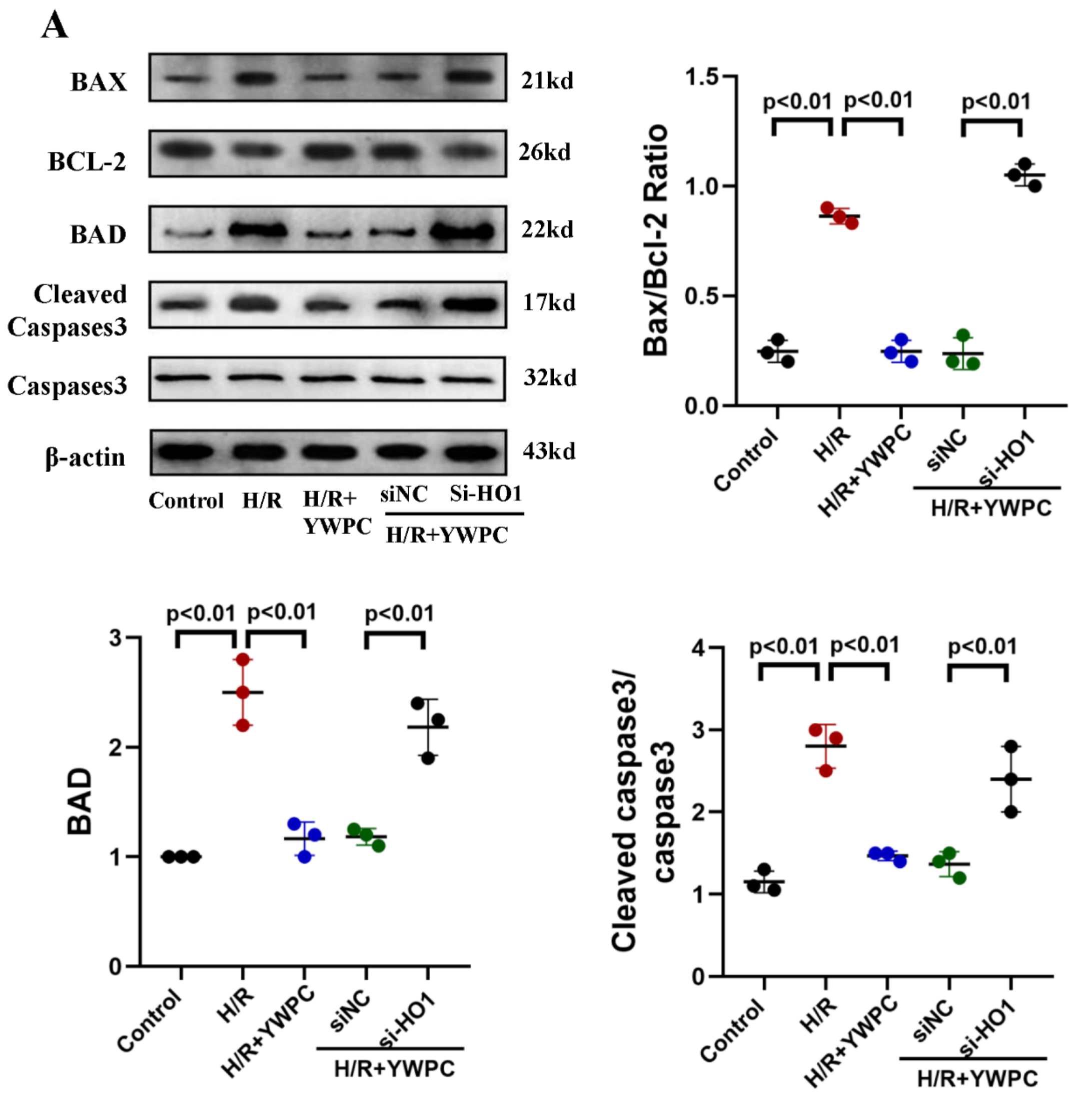

Supplementary Figure 2: Effect of HO1 Silencing on the Myocardial Protective Effects of YWPC. (A) Western blot analysis of the relative protein expression of BAX, BCL-2, BAD, Cleaved Caspase-3 and Caspase-3 in vitro. One-way ANOVA was used for comparisons among four groups, followed by Tukey's post hoc analysis.
