# Supplementary material for: Yellow Wine Polyphenolic Compounds protect against myocardial ischemia-reperfusion injury in rats by activating Nrf2 nuclear translocation to regulate the balance of mitochondrial fission and fusion
Source: Front Cardiovasc Med. 2025 May 23;12:1506388. doi: 10.3389/fcvm.2025.1506388 (PMC12141274; doi:10.3389/fcvm.2025.1506388)

Figure 2

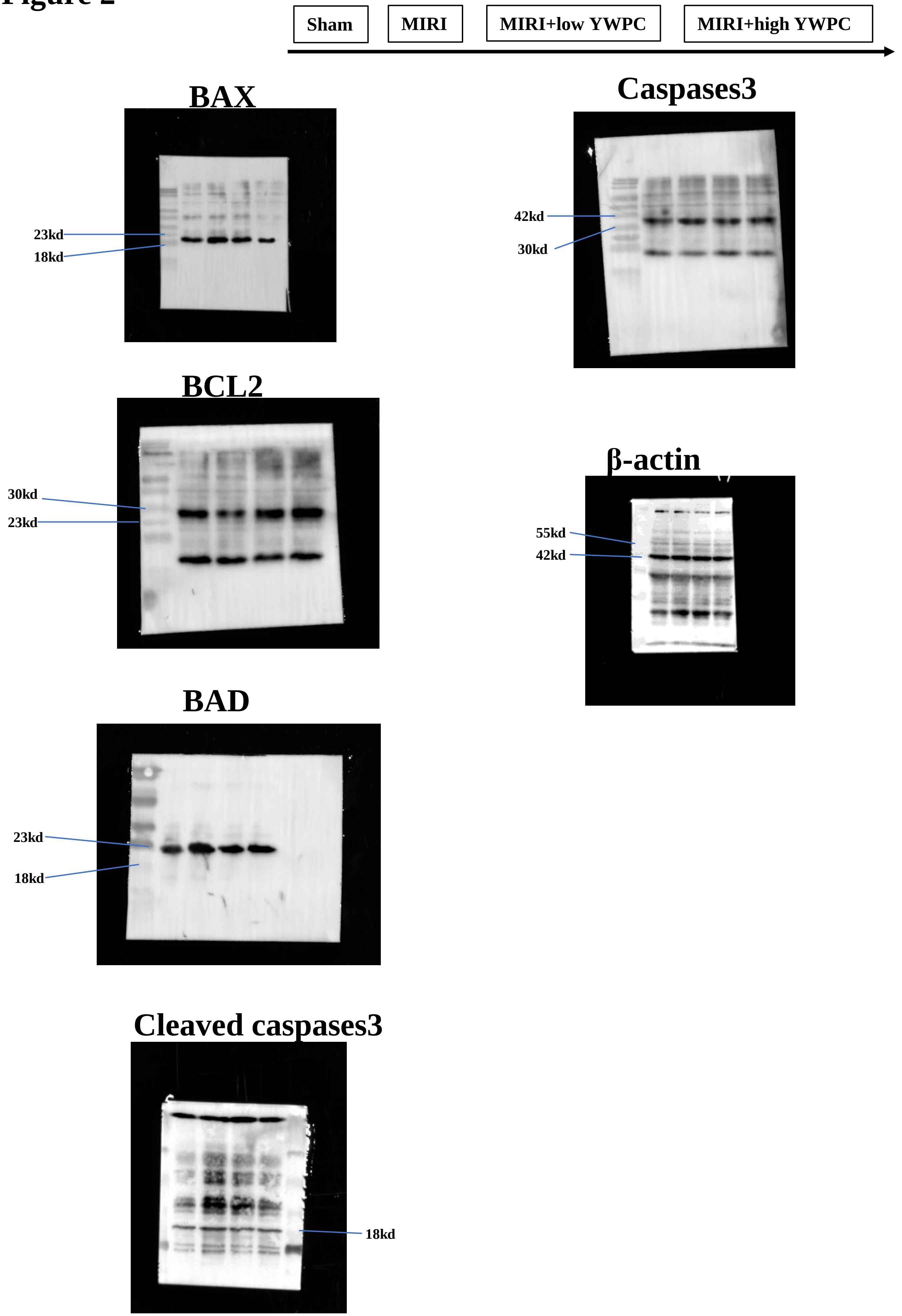

Sham
MIRI
MIRI+low YWPC
MIRI+high YWPC
BAX
23kd
18kd
Caspases3
42kd
30kd
BCL2
30kd
23kd
β-actin
55kd
42kd
BAD
23kd
18kd
Cleaved caspases3
18kd

Figure 3

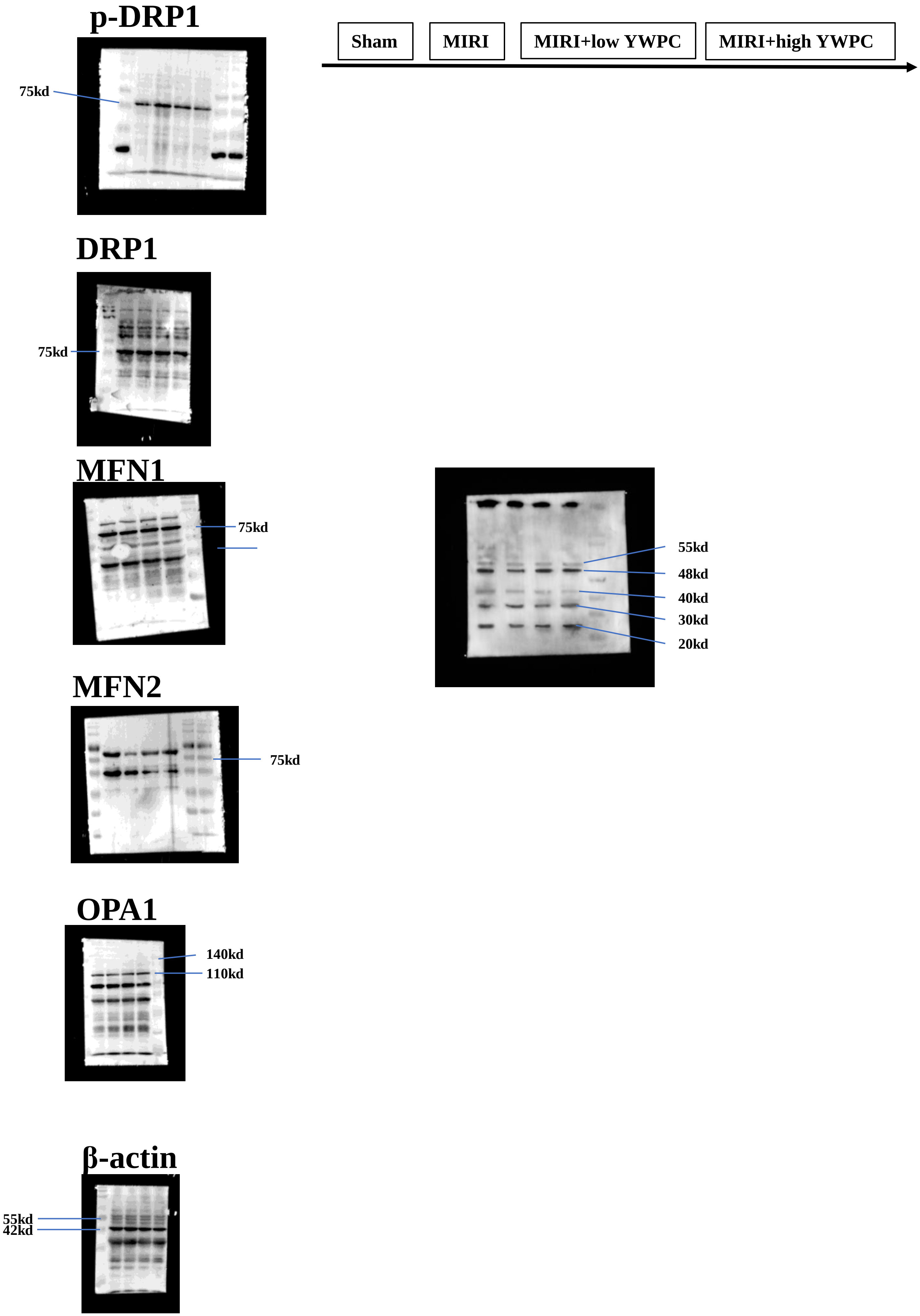

p-DRP1
75kd
Sham
MIRI
MIRI+low YWPC
MIRI+high YWPC
DRP1
75kd
MFN1
75kd
55kd
48kd
40kd
30kd
20kd
MFN2
75kd
OPA1
140kd
110kd
β-actin
55kd
42kd

Figure 5

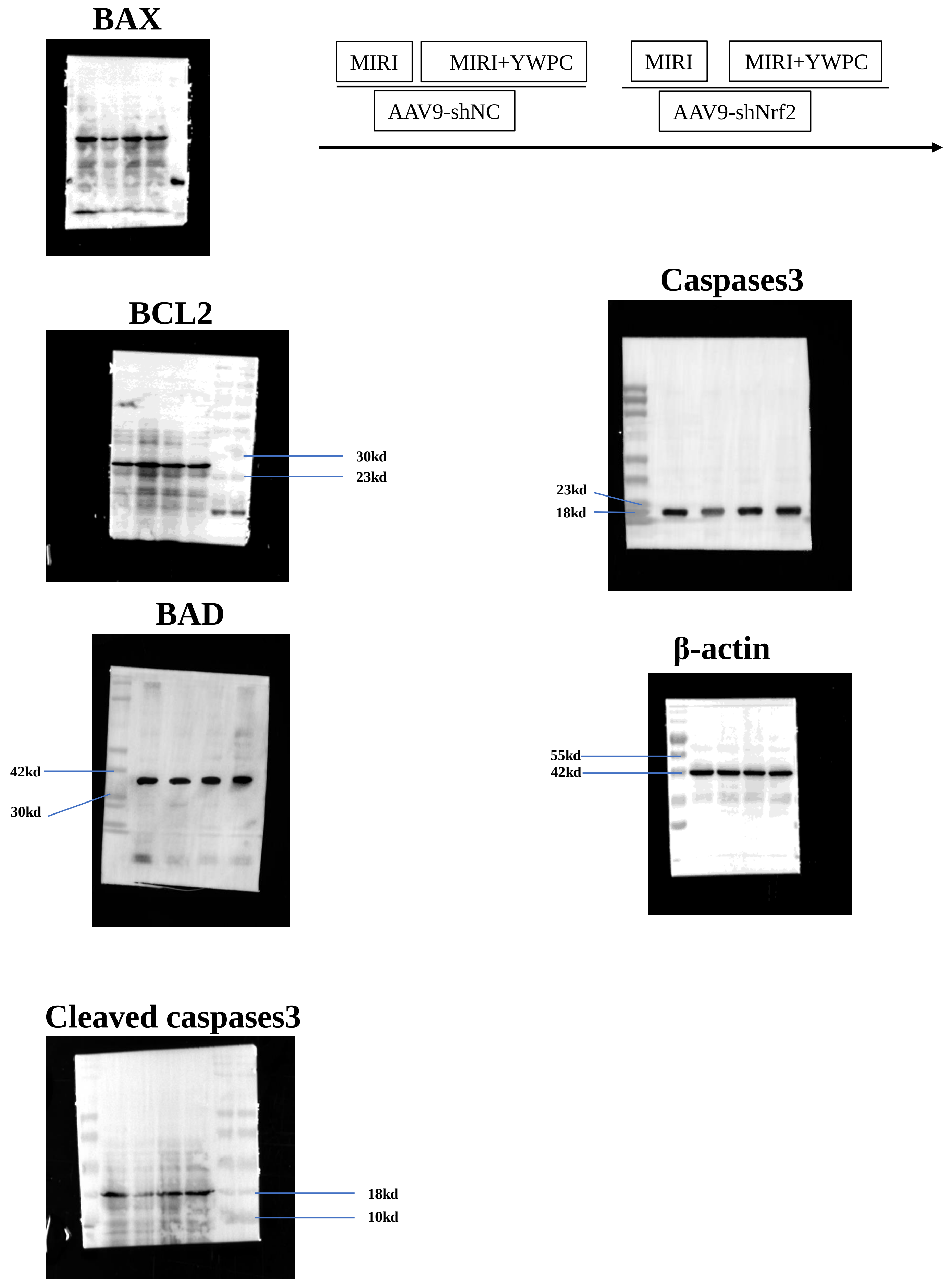
BAX
MIRI
MIRI+YWPC
MIRI
MIRI+YWPC
AAV9-shNC
AAV9-shNrf2
BCL2
30kd
23kd
Caspases3
23kd
18kd
BAD
42kd
30kd
β-actin
55kd
42kd
Cleaved caspases3
18kd
10kd

Figure 6

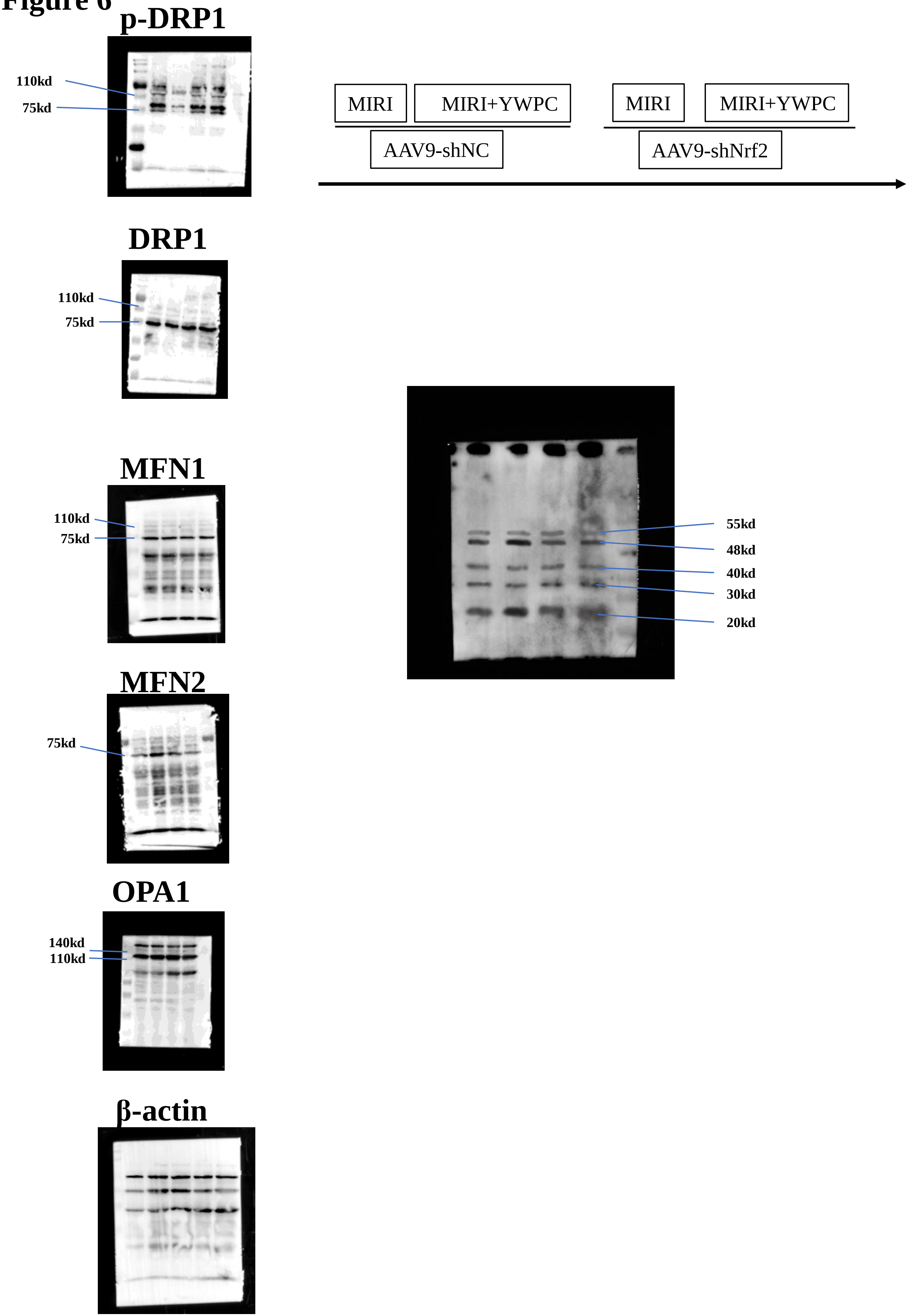
p-DRP1
110kd
75kd
MIRI
MIRI+YWPC
MIRI
MIRI+YWPC
AAV9-shNC
AAV9-shNrf2
DRP1
110kd
75kd
MFN1
110kd
75kd
55kd
48kd
40kd
30kd
20kd
MFN2
75kd
OPA1
140kd
110kd
β-actin

Figure 7

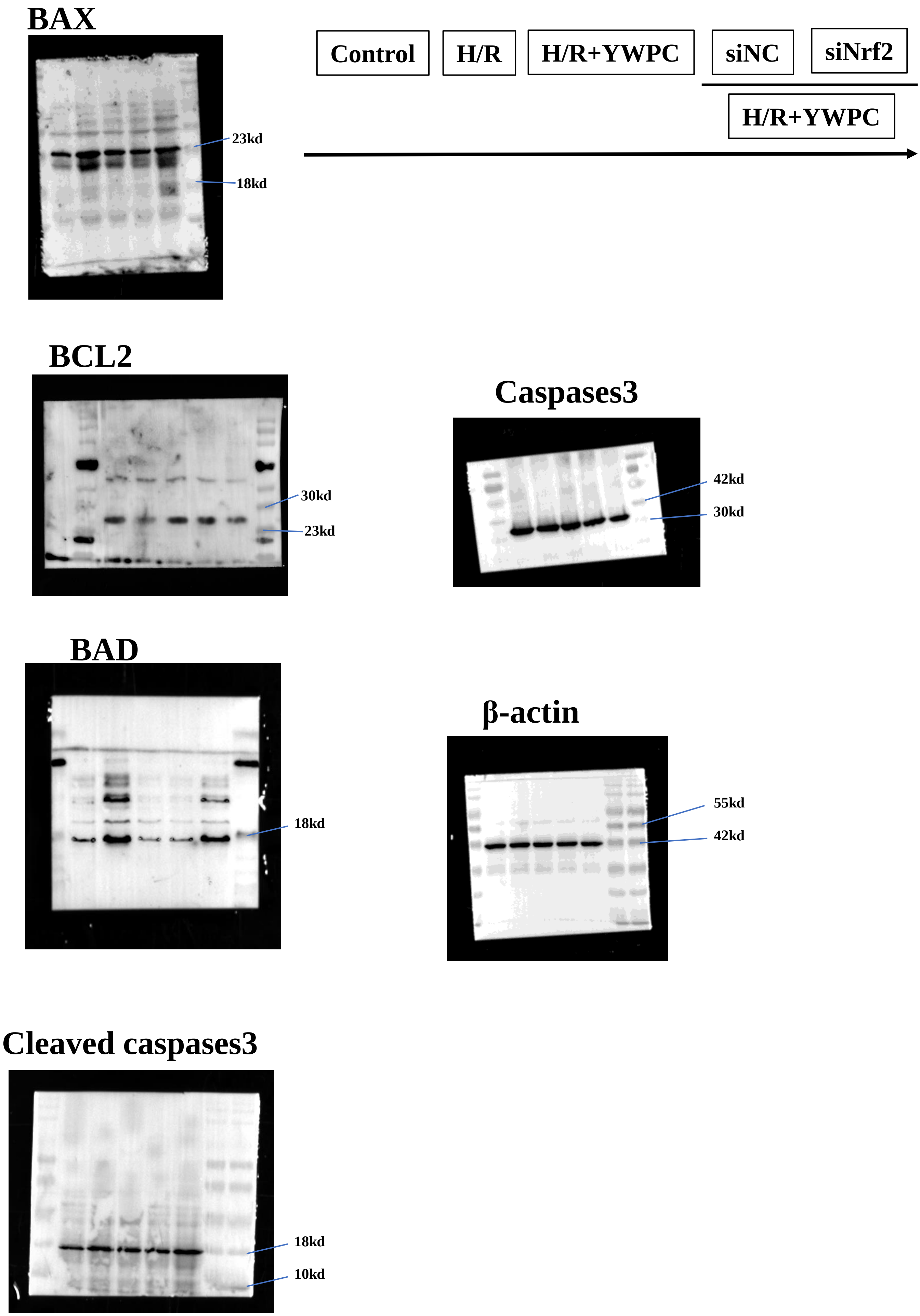

BAX
23kd
18kd
Control
H/R
H/R+YWPC
siNC
siNrf2
H/R+YWPC
BCL2
30kd
23kd
Caspases3
42kd
30kd
BAD
18kd
β-actin
55kd
42kd
Cleaved caspases3
18kd
10kd

Figure 8

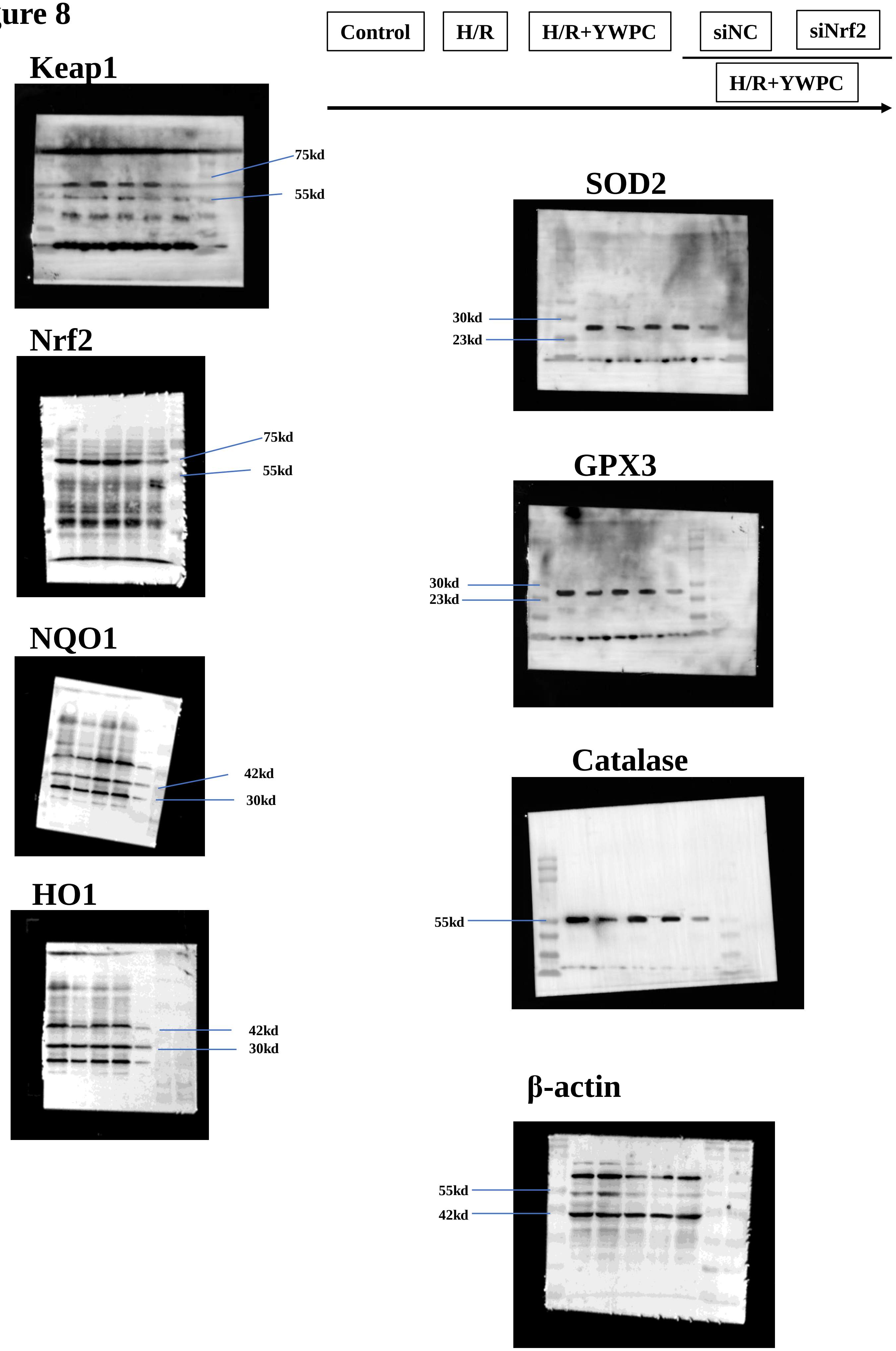

Control
H/R
H/R+YWPC
siNC
siNrf2
H/R+YWPC
Keap1
75kd
55kd
Nrf2
75kd
55kd
NQO1
42kd
30kd
HO1
42kd
30kd
SOD2
30kd
23kd
GPX3
30kd
23kd
Catalase
55kd
β-actin
55kd
42kd

Figure 10

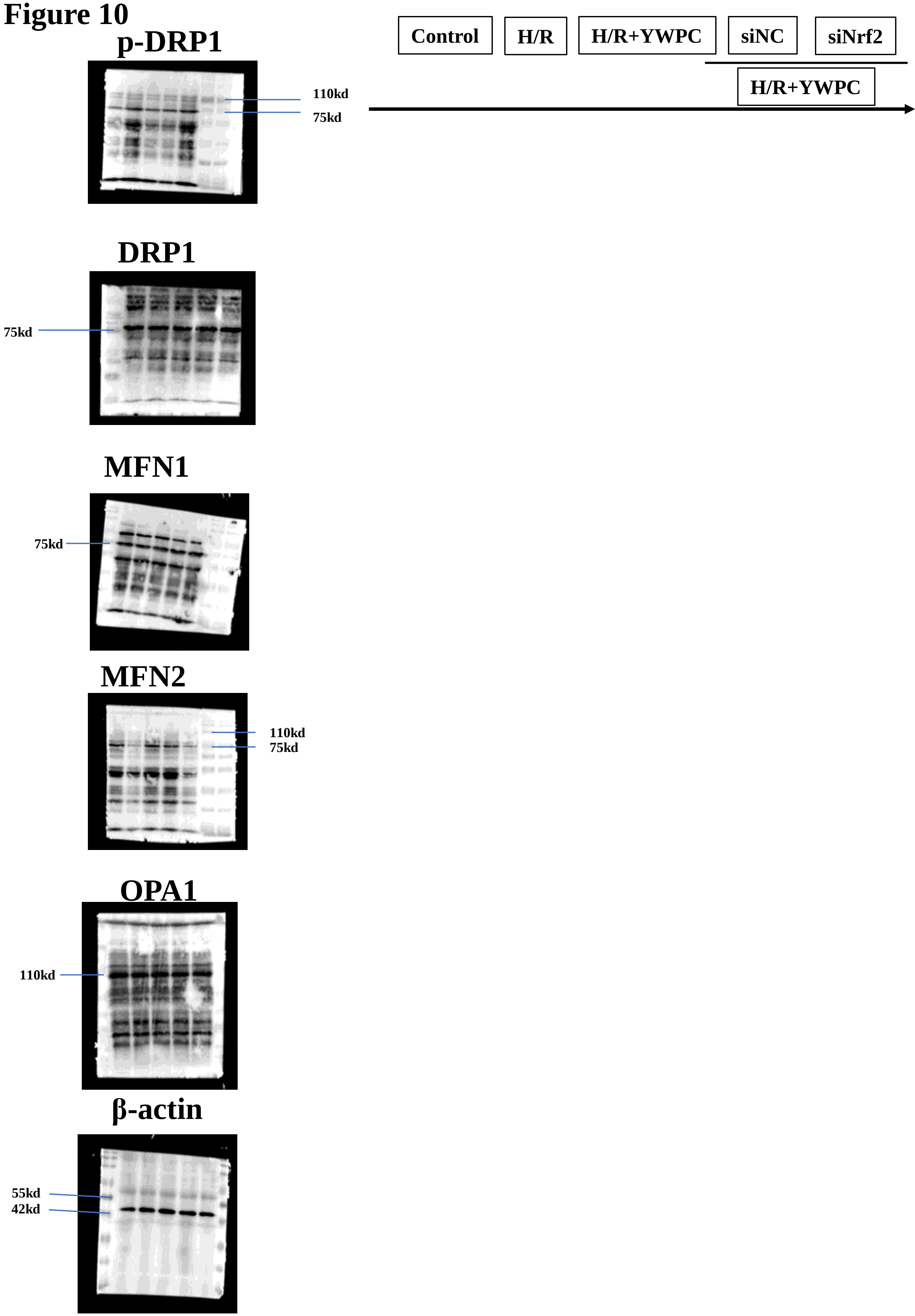

p-DRP1
Control
H/R
H/R+YWPC
siNC
siNrf2
H/R+YWPC
110kd
75kd
DRP1
75kd
MFN1
75kd
MFN2
110kd
75kd
OPA1
110kd
β-actin
55kd
42kd

Supplementary Figure 1

Nrf2

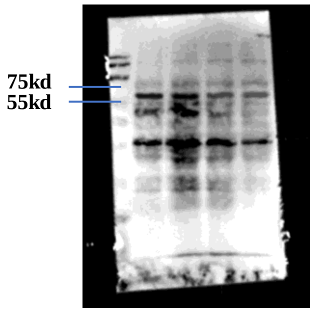

Control① Control ② AAV9-shNrf2① AAV9-shNrf2②

β-actin

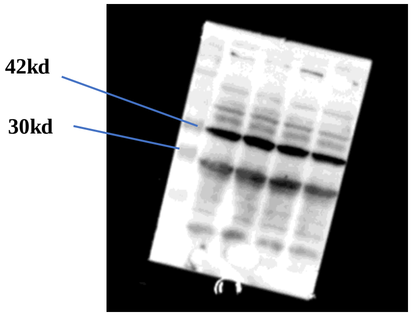

Supplementary Figure 2

Control | H/R | H/R+YWPC | siNC | Si-HO1

(siNC and Si-HO1: H/R+YWPC)

BAX

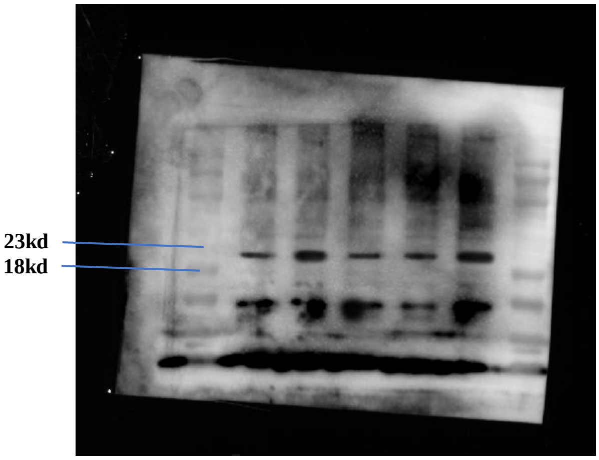

BCL2

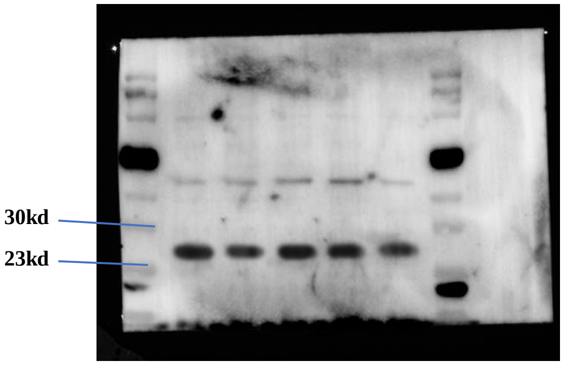

Caspases3

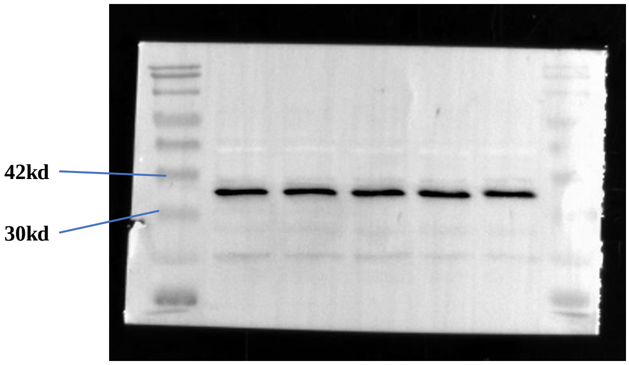

BAD

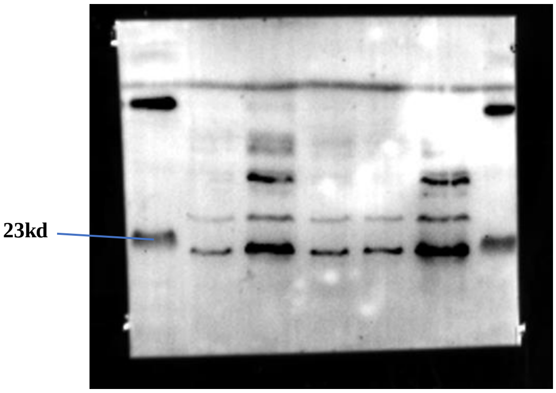

β-actin

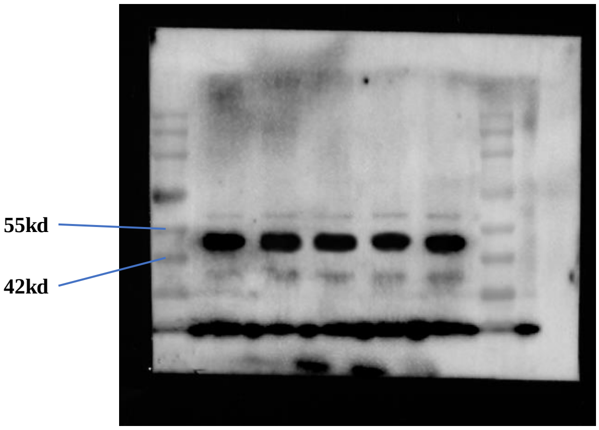

Cleaved Caspases3

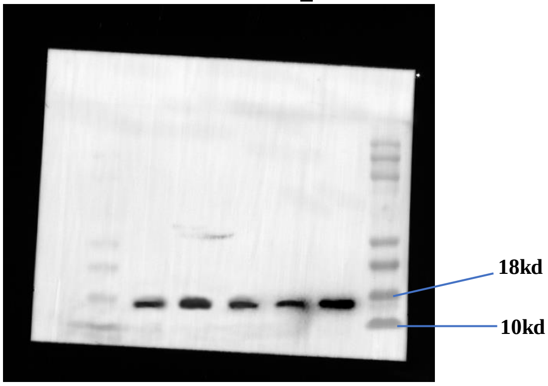

Supplement: Supplementary file 2 [file Datasheet2.pdf]
